# High thrombin activity associated with crohn’s disease induces microbiota pathogenicity contributing to mucosal inflammation

**DOI:** 10.1080/19490976.2026.2687903

**Published:** 2026-06-17

**Authors:** Guillaume Le Cosquer, Melissa Pannier, Julie Thevenin, Laura Guiraud, Melissa David, Alexia Dumas, Elodie Meunier, Perrine Rousset, Simone Palese, Carmine Giorgio, Louis Buscail, Barbara Bournet, Cyrielle Gilletta, Adrian Culetto, Cindy Canivet, Etienne Buscail, Caroline Camaré, Laurent Alric, Céline Deraison, Nathalie Vergnolle, Jean-Paul Motta

**Affiliations:** a University of Toulouse, INSERM, INRAe, ENVT, Institute of Digestive Health Research (IRSD), Toulouse, France; b Université of Toulouse, Toulouse University Hospital (CHU Toulouse), Hôpital Rangueil, Department of Gastroenterology and Pancreatology, Toulouse, France; c Université de Toulouse, Toulouse University Hospital (CHU Toulouse), Hôpital Rangueil, Department of Colorectal Surgery, Toulouse, France; d Université de Toulouse, Toulouse University Hospital (CHU Toulouse), Hôpital Rangueil, Department of Clinical Biochemistry, Toulouse, France; e Université de Toulouse, Toulouse University Hospital (CHU Toulouse), Hôpital Rangueil, Department of Internal Medicine and Clinical Immunology, Toulouse, France; f Department of Pharmacology-Physiology, Institut de Pharmacologie de Sherbrooke, Faculty of Medicine and Health Sciences, Université de Sherbrooke, Sherbrooke, QC, Canada

**Keywords:** Crohn's disease, thrombin, host–microbiota interactions, dysbiosis, biofilms, mucosal inflammation, Toll-Like Receptor 5, fecal biomarkers

## Abstract

Thrombin is a serine protease produced by the intestinal epithelium that contributes to mucosal homeostasis by limiting microbial encroachment. In Crohn's disease, mucosal thrombin activity is markedly increased, suggesting a role in disease pathophysiology. We hypothesized that excessive luminal thrombin disrupts host–microbiota interactions and promotes a pathogenic microbial phenotype. Thrombin levels were significantly elevated in fecal samples from a subset of Crohn's disease patients compared with healthy controls. *Ex vivo* experiments using human colonic biopsy-derived microbiota, cultured as polymicrobial biofilms, revealed that thrombin exposure disrupted biofilm integrity, enriched the protein-rich extracellular matrix, and promoted bacterial dispersal. Dispersed bacteria displayed enhanced adhesion to intestinal epithelial cells and triggered inflammatory and antimicrobial responses through Toll-Like Receptor 5 signaling. Although the global taxonomic composition showed limited changes, metatranscriptomic analyses demonstrated distinct microbial activity profiles in the presence of thrombin. *In vivo*, intracolonic thrombin altered mucosal biofilm organization in mice, and the transfer of thrombin-exposed mucosal microbiota to germ-free recipients induced mucosal inflammation and bacterial translocation. Finally, in a rat model of colitis, pharmacological inhibition of thrombin activity restored mucosal biofilm architecture and reduced tissue damage. These findings identify thrombin as a host-associated modulator of microbial functional dysbiosis in patients with Crohn's disease. By driving a shift toward a pathogenic microbial phenotype, excessive thrombin links epithelial activity with altered host–microbiota interactions and mucosal inflammation. Thrombin measurement in fecal samples may represent a non-invasive marker of microbiota pathogenicity, and therapeutic targeting of thrombin offers a promising complementary strategy to immune-directed treatments in Crohn's disease.

## Graphical abstract

**Figure:**
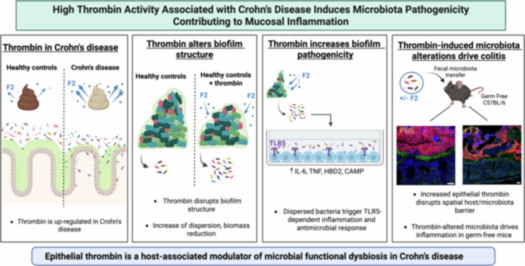

## Introduction

Crohn’s disease (CD) is a chronic, lifelong condition characterized by persistent inflammation of the gastrointestinal tract.^1^ While the exact pathophysiology of CD remains incompletely understood, it is widely accepted that the disease arises from a complex interplay of genetic, environmental, and immunological factors. Recent studies have suggested that proteolytic dysregulation may be a contributing factor.^2^ Indeed, elevated levels of proteolytic activity have been observed in tissue samples from patients with inflammatory bowel disease (IBD).^3-6^ Remarkably, Denadai-Souza et al. observed that Thrombin was the most active serine protease, with a 100-fold increased activity in the mucosa of CD patients compared to healthy controls.^3^ Further, Motta et al. demonstrated that the intestinal epithelium is a source of active thrombin production in the intestinal lumen.^7^ Such high thrombin activity in the mucosa of CD patients likely impacts mucosal and microbiota homeostasis.

In previous work, we have demonstrated that increased local activity of thrombin leads to mucosal damage and inflammation through mechanisms involving the activation of host protease-activated receptors (PAR)-1 and PAR-4.^4^ However, the effects of increased thrombin activity on mucosa-associated microbiota, its taxonomy, behavior, and interactions with the underlying mucosal tissue are unknown. It is well documented that in CD patients, environmental changes contribute to gut dysbiosis.^8^ This dysbiosis is characterized by reduced microbial diversity and the expansion of pathobionts, which can trigger inflammatory responses in the intestinal epithelium. We hypothesized that thrombin might be one of the environmental factors (originating from the host) that contribute to microbiome dysregulation and dysbiosis.

## Materials and methods

### Human clinical data and samples

Colonic biopsies and feces were collected with informed consent from the CAPITOL (2021–2023, ID RC31/21/0038) and COLIC (2017–2020, DC-2015-2443) cohorts at Toulouse University Hospital (Supplementary Figure 1). Donors with recent (≤3 months) use of antibiotics, probiotics, prebiotics, anti-thrombin drugs, or a history of IBS/colorectal cancer were excluded. Biopsies came from healthy subjects undergoing colonoscopy for polyp screening (*N* = 10) or positive fecal tests (*N* = 1). Feces were collected from CD (*N* = 37), ulcerative colitis (UC) (*N* = 9), and non-IBD controls (*N* = 9). The clinical characteristics (body mass index (BMI), smoking, and Charlson index) are shown in Supplementary Tables 1 (colonic biopsies donors for biofilm cultures), 2 (stool donors), and 3 (colonic biopsies for thrombin activity assessment).

### Thrombin quantification

Protein extraction and thrombin quantification in stool followed published protocols.^7^ Reference proteins included thrombin (52 ng, 70 nM), prothrombin (58 ng, 157 nM), and PRSS3 (2 nM)-cleaved thrombin (265 nM). Band intensities were analyzed using ImageLab (Bio-Rad), and the results are expressed as pg thrombin/µg protein (Supplementary Figure 2). Thrombin activity was assessed in biopsy supernatants after 1 h incubation in HBSS as previously described.^3^ ^,^ ^7^

### Ex vivo biofilms and phenotyping

Mucosa-associated biofilms were grown from biopsies in supplemented BHI using MBEC™ devices (Innovotech, Canada) at OD_600_ = 0.1.^7^ ^,^ ^9-11^ After 72 h, biofilms were treated for 24 h with thrombin (0–10 U/mL). Biomass (safranin-O), viability (resazurin), and matrix (WGA-GFP, Bodipy SYPRO) were assessed as previously described.^7^ ^,^ ^9-11^ Dispersed bacteria were collected for 16S sequencing and 4 h coculture with HT29-MTX/Caco-2 (3:1, OD_600_ = 0.01).^11^ All steps were performed anaerobically (Don Whitley A20). All conditions were tested in  ≥ 4 replicates; LF82 *E. coli* served as a control. The same experimental approach was applied to additional adherent-invasive E. coli strains, including the LF82 wild-type strain, its isogenic ΔfliC mutant (lacking the *fliC* gene encoding flagellin), the NRG 857c strain, as well as *E. coli*/*Enterobacteriaceae* isolates obtained from patient-derived mucosal microbiota using RAPID'E.coli2 (Biorad, Hercules, USA).

### qRT-PCR (human and animal samples)

RNA was extracted using TRIzol (Euromedex), reverse-transcribed (Maxima First Strand Kit), and amplified by SYBR Green qPCR (Takyon, Eurogentec) on a LightCycler, according to manufacturers’ instructions. The expression levels were normalized to both *HPRT* and *GAPDH* genes using 2-ΔΔCT. For rats, RNA was extracted from the distal colon, homogenized with Precellys, and qPCR run on a BioMark (Fluidigm). The primer list is provided in Supplementary Tables 4 and 5.

### TLR5 assays

HEK-Blue™ hTLR5 cells (InvivoGen, France) were cultured per manufacturer’s instructions at 37 °C and 5% CO₂. After 4 h of coculture with biofilm-dispersed bacteria, the supernatants were added to HEK-Blue™ detection medium, and the responses were normalized to those of thrombin-free controls. For the inhibition assays, Caco-2:HT29 (3:1) cells were pretreated for 1 h with an anti-TLR5 antibody (1  µg/mL, InvivoGen) before interaction.

### TNBS induced colitis

Male Wistar rats (250–300 g, Janvier Labs, France) were fasted and given trinitrobenzene sulfonic acid (TNBS) intracolonically to induce colitis as described in ^4^ (Supplementary Figure 1). Three groups (control Phosphate Buffer Saline (PBS), TNBS in 0.1% DMSO, and TNBS + dabigatran [0.1 µg/kg] in 0.1% DMSO) received 4 days of intracolonic treatment. Distal colons were harvested and methacarn-fixed for fluorescent in situ hybridization (FISH).

### Fluorescent In situ hybridization (FISH)

Methacarn-fixed colonic tissue was paraffin-embedded and hybridized with an EUB338-Cy3 16S rRNA probe (Eurofins). The DNA was counterstained with DAPI and polysaccharides with fluorescein-labeled WGA (Thermo Fisher). Biofilm damage was scored in ≥3 fields/mouse following previously published criteria.^7^ Images were obtained on a Leica LSM 710 and FIJI (v1.51).

### *In vivo* microbiota inoculation

C57BL/6 male mice (8 weeks old) were first exposed intracolonically for 10 d to active thrombin (5 or 20 U/day; Sigma T-6884) or saline under isoflurane (Supplementary Figure 1). The whole colon mucosa was scraped, the microbiota was recovered, and the samples were resuspended in PBS/20% glycerol (OD600 = 1.5). Handling was performed under anaerobic conditions (Don Whitley A20). Germ-free C57BL/6 mice (Janvier, 8 weeks, CREFRE axenic facility) were grouped into true germ-free controls (*n* = 2), vehicle (*n* = 5), 5 U (*n* = 7), and 20 U (*n* = 8) (Supplementary Figure 1). All groups except the GF group received 200  µL mucosa-microbiota slurry via gavage. After 4 d, the colons were scored (Wallace scale^12^), fixed, and the feces were frozen at –80 °C. Lymph nodes/spleens were plated on blood agar to assess translocation. The entire protocol was approved by the French Ethics Committee (APAFIS 2021051116031549).

## 16S rRNA gene amplicon sequencing and shotgun metatranscriptomics

DNA from feces or biofilm bacteria was extracted (EZNA kit, VWR). The 16S V3–V4 regions (341F/806R) were sequenced on an Illumina MiSeq (2 × 250 bp, Biomnigene, Besançon, France). The reads were processed using QIIME2 (2024.10): primer trimming (Cutadapt), denoising (DADA2 v1.30.0), and taxonomy assignment (SILVA 138.2, classify-sklearn). PICRUSt2 (v2.5.1) inferred the enzyme class (EC), MetaCyc, and KEGG pathways from ASVs using GTDB r214 + MinPath.^13^ Shotgun metatranscriptomics was performed on CAPITOL cohort biofilm bacteria with healthy controls (HC; ± 10 U/mL thrombin, *n* = 5) and CD patients (*n* = 5). RNA extraction followed low-biomass protocols (GenoScreen, Lille, France), and libraries were prepared with TruSeq Stranded mRNA and sequenced (Illumina, ~15M reads/sample, GenoScreen). Data were processed on Nephele (NIH) via biobakery workflows (v3.0.0.a.7), including KneadData v0.10.0, MetaPhlAn v3.0.7, and HUMAnN v3.0.0.alpha. Downstream analyses and visual outputs were then performed in R.

### Statistical analysis

Statistical analyses were performed using GraphPad Prism v10 and R v4.3.0. Continuous variables are presented as mean ± SD; categorical variables as proportions. Correlations were tested by Spearman’s method. Grubbs’ test removed outliers. Group comparisons used ANOVA (Dunnett correction) or Kruskal–Wallis as appropriate. Microbial data were processed in R/QIIME2. Beta-diversity was analyzed with PCoA/PCA (phyloseq); PERMANOVA tested group differences (pairwise Adonis, 9,999 permutations). LEfSe, microbiomeMarker, and DESeq2 were used for differential abundance with Benjamini–Hochberg correction. PLS-DA was run using the mixOmics package (6.26). Throughout the paper, statistical significance was set at *P* < 0.05.

## Results

### CD is associated with increased fecal thrombin

**Figure 1.**
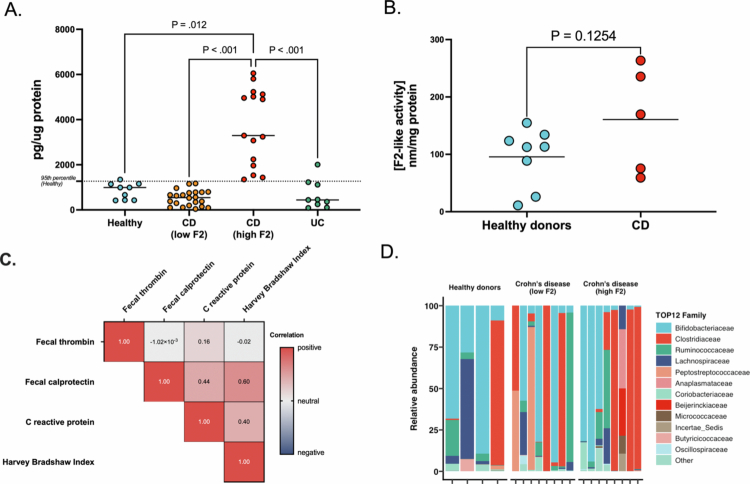
Thrombin activity is increased in Crohn’s disease (CD) patients independently of the inflammation status and microbiota taxonomy. (A) The study groups include healthy individuals (*n*=9), CD patients with fecal thrombin levels above the 95th percentile of healthy controls (high F2, *n*=15), CD patients with levels below this threshold (low F2, *n*=22), and patients suffering from ulcerative colitis (UC, *n*=9). The scatter plot depicts the quantity of fecal thrombin (determined by Western blot analysis with a specific anti-thrombin antibody, and expressed as pg/µg total protein amount) for each individual and was grouped for every four cohorts of individuals. The full line corresponds to the mean value of each cohort. The dotted line represents the 95th percentile of healthy controls. Statistical significance was determined by Brown–Forsythe and Welch ANOVA tests, where *P *< 0.05 was considered significant. (B) The study groups include colonic biopsies from healthy individuals (*n*=8) and individuals with CD (*n*=5). The scatter plot depicts the quantity of thrombin activity (determined by the VPR-AMC assay with pre-incubation of samples with dabigatran) for each individual. Statistical significance was determined by Brown–Forsythe and Welch ANOVA tests, where *P*<0.05 was considered significant. (C) Heatmap represents Spearman's correlation coefficients between thrombin, clinical (Harvey Bradshaw Index), blood (CRP), and fecal (Calprotectin) disease activity surrogate markers from 33 human donors with CD (4 individuals were excluded because of incomplete dataset, e.g., unavailable fecal calprotectin or C-reactive protein blood levels). The parameters included were considered as continuous features. The color gradient from blue to red signifies negative to positive correlations. There were no significantly positive or negative correlations (*P*<0.05). (D) Microbiota from fecal samples were cultured overnight in BHI under anaerobic conditions prior to DNA extraction to assess taxonomic differences between healthy individuals (*n*=4), CD patients with high (high F2, *n*=8) and low fecal thrombin levels (low F2, *n*=8). The bar plots illustrate the relative abundance of the TOP12 families according to each individual.

We analyzed stool samples from HC (*N* = 9), CD patients (*N* = 37), and UC patients (*N* = 9). Sociodemographic and disease-specific characteristics are detailed in Supplementary Table 2. Fecal sample western blot analysis revealed marked heterogeneity, identifying a distinct subset of CD patients with elevated thrombin levels. Using the 95th percentile of HC as a threshold, CD patients were stratified into two subgroups: those with thrombin levels above this range (high F2, *N* = 15) and those below this range (low F2, *N* = 22). Fecal thrombin levels were significantly increased in high-F2 CD patients compared with HC (*p* = 0.01), UC patients (*p* < 0.001), and low-F2 CD patients (*p* < 0.001; Figure 1A and Supplementary Figure 2).

To document the elevated thrombin activity at the mucosal level, we assessed thrombin activity in colonic biopsy supernatants from 5 randomly selected CD patients and 8 HC (with a personal or familial history of polyps) from a cohort where biopsies were performed (the CAPITOL cohort), revealing a similar trend with subgroups of CD patients having high thrombin activity in their colonic tissues and others having a low thrombin activity (Figure 1B). Sociodemographic and disease-specific characteristics for these subjects are detailed in Supplementary Table 3.

### High levels of fecal thrombin in CD is independent of disease activity and fecal microbiota taxonomy but reveals a distinct microbiome functional profile

We found no significant correlation between thrombin levels detected in fecal samples and systemic inflammation (serum C-reactive protein (CRP)), gut-specific inflammation (fecal calprotectin), or clinical activity (Harvey-Bradshaw index) among CD samples, suggesting that thrombin elevation occurs independently of disease activity (Figure 1C).

**Figure 2.**
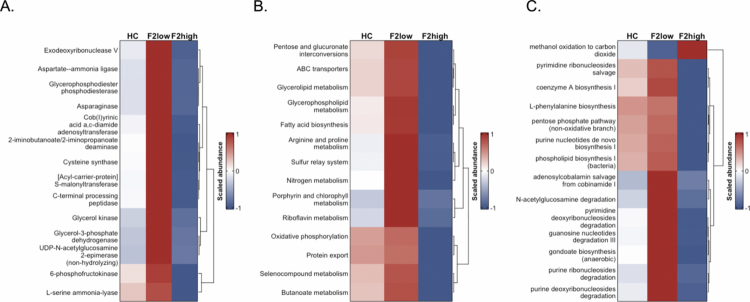
Predicted functional profiling of stools from healthy controls (HC) and Crohn’s disease (CD) patients. Total DNA was sequenced using Illumina (V3–V4 region) from the stools of healthy controls (HC, *n* = 4), CD patients with fecal thrombin levels below the 95th percentile of healthy controls (F2low, *n*=8), and CD patients with levels above this threshold (F2high, *n*=8). (A–C) Scaled abundance heatmaps of (A) enzyme classes (EC), (B) KEGG pathways, and (C) MetaCyc pathways assigned for each group (HC, HC+F2, and CD) and compared with Kruskal–Wallis (*P*<0.05). In all panels, color gradients from blue (low abundance) to red (high abundance) indicate scaled relative pathway expression across samples.

We then compared the taxonomic composition of cultured stool samples from 4 randomly selected HC, 8 patients from the high F2 CD group, and 8 patients from the low F2 CD group. No significant taxonomic shifts were observed between the high- and low-F2 groups at the family and phyla levels (Figure 1D). Additional comparisons, including taxa abundance, alpha and beta diversity, and differentially abundant taxa identified by LEfSe (Linear discriminant analysis Effect Size), are provided in Supplementary Figures 3–5. As widely reported in the literature, we observed a reduction in overall alpha diversity in CD patients compared to HC (Supplementary Figure 4A). Predicted functional profiles, using the PICRUSt2 tool, revealed distinct microbial metabolic patterns across the groups. Focusing on the 15 most statistically significant features (Kruskal‒Wallis, *P* <  0.05; Figure 2), we identified several differences in microbial metabolic functions. The CD high-F2 group showed reduced abundance of enzymatic functions linked to 6-phosphofructokinase and L-serine ammonia-lyase, while the low-F2 group was enriched in cysteine synthase and C-terminal processing peptidase (Figure 2A). The low-F2 group also had higher levels of KEGG pathways, including protein export and sulfur relay systems (Figure 2B), as well as MetaCyc pathways linked to purine degradation and anaerobic metabolism (Figure 2C).

Elevated mucosal and fecal thrombin levels define a distinct subgroup of CD patients, independently of disease activity, and may influence gut microbiota function and behavior.

### CD-associated high thrombin activity impacts intestinal microbial community *in vitro* and *in vivo*

**Figure 3.**
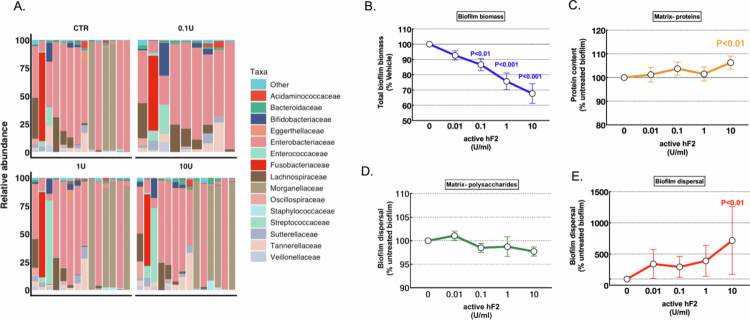
High thrombin exposure is associated with physical alterations of biofilms without a significant taxonomic shift. Mucosa-associated microbiota (isolated from colonic biopsies) from healthy individuals (*n *= 11) were cultured in vitro as a polymicrobial anaerobic biofilm and exposed to increasing concentrations of human purified thrombin (ranging from 0 to 10 U/mL). (A) The bar plots illustrate the relative abundance of the 15 major taxa according to each condition. (B) The total biofilm biomass, (C) the total protein content of extracellular biofilm matrix, and (D) the total sugar content of extracellular biofilm matrix were measured. (E) The rate of biofilm-dispersed bacteria was measured during a 24-hour period of culture. For each individual, the raw values were normalized to the average values of the condition without thrombin. The plot represents the mean values for all individuals in each condition (human purified thrombin concentrations ranging from 0 to 10 U/mL). Statistical significance was determined by ANOVA followed by Dunnett’s test for multiple comparisons, where *P*<0.05 was considered significant.

To explore the effect of high thrombin levels on the biofilm organization, we cultured the microbiota from colonic biopsies of healthy individuals (clinical characteristics summarized in Supplementary Table 1) on a MBEC biofilm device reproducing the tissue-associated polymicrobial anaerobic biofilm phenotype.^7^ ^,^ ^9^ ^,^ ^10^ These biofilms were then exposed to increasing concentrations of human purified thrombin (ranging from 0 to 10 U/mL). The highest dose of thrombin used was calculated according to the highest amount of thrombin that was measured in the fecal samples of high-F2 CD patients in Figure 1. Using 16S V3‒V4 metabarcoding, we first identified that the microbial community within these biofilms was predominantly dominated by *Clostridia, Gammaproteobacteria,* and *Actinomycetes* at the class level, which is consistent with previous data on mucosa-associated microbiota in healthy individuals.^14^ In terms of relative abundance at the taxa level, we observed no significant shift following thrombin exposure *in vitro* (Figure 3A). Additional comparisons, including taxa abundance, alpha and beta diversity, and differentially abundant taxa identified by LEfSe (Linear discriminant analysis Effect Size), are provided in Supplementary Figures 6 and 7.

Next, we characterized the physical structure of these *ex vivo* biofilms and found a significant decrease in total biofilm biomass after exposure to high thrombin levels (Figure 3B; *p* < 0.01). Notably, biofilms exposed to 10  U/mL of human thrombin exhibited an increased protein matrix composition compared to other groups (Figure 3C; *p* < 0.01), while no change was observed in sugar matrix content (Figure 3D). We assessed the ability of these *ex vivo* biofilms to disperse bacteria, considering that previous studies have suggested that dispersed bacteria represent a more pathogenic stage (distinct from both biofilm and planktonic stages) and that high dispersal is a virulent trait that increases contact between pathobionts and the underlying epithelium.^9^ ^,^ ^10^ ^,^ ^15^ Biofilms exposed to high thrombin levels showed a significantly higher dispersal rate compared to unexposed biofilms (Figure 3E; *p* < 0.01).

*In vivo* daily intracolonic administration to mice of 5 U/mL of thrombin, a concentration in the range of the activity that is detected in tissues from CD patients^3^ revealed significant mucosal biofilm structure damage, associated with minor taxonomic changes, thereby echoing *in vitro* observations and confirming the effects of high thrombin on biofilm disorganization *in vivo* (Supplementary Figure 8, 9 and 10).

Taken together, these results suggest that high thrombin exposure, a feature associated with CD, induces substantial alterations in biofilm architecture, leading to increased bacterial dispersion and infiltration into colonic tissue.

### Thrombin is a major contributor to colitis-induced alterations of microbial biofilm architecture

We used a TNBS-induced experimental colitis model in rats, in which thrombin activity is known to be upregulated.^4^ We compared the spatial organization of microbiota biofilm using FISH across three groups: animals treated intracolonically with vehicle (PBS, *n* = 5), animals treated with TNBS alone (*n* = 5) and animals treated with both TNBS and dabigatran (a thrombin inhibitor, *n* = 4).

**Figure 4.**
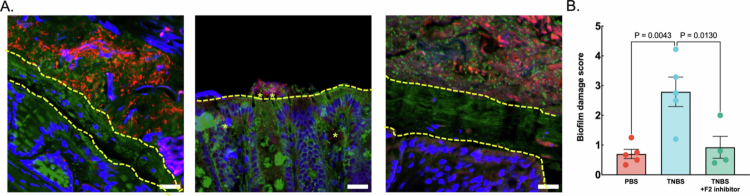
Inhibition of thrombin prevents colitis-induced biofilm disorganization in vivo. Using 16S rRNA fluorescent in situ hybridization (FISH), we visualized the tissue-associated microbiota in the distal colon of Wistar rats (150 g) intracolonically exposed to 4 days of 0.1% DMSO (vehicle) with PBS intracolonic administration at day 1 (*n*= 5); intracolonically exposed to 4 d of 0.1% DMSO (vehicle) with TNBS intracolonic administration at day 1 (*n*=5); and intracolonically exposed to 4 days of 0.1 µg/kg Dabigatran (a thrombin inhibitor) with TNBS intracolonic administration at day 1 (*n*=4, right panel ). (A) Representative images (PBS, left panel; TNBS, middle panel; TNBS + thrombin inhibitor, right panel) are presented with blue is DAPI staining for host nuclei, green is fluorescein-coupled wheat germ agglutinin for sugar-rich content (e.g., mucus layer), and red is the 16S-Cyanine3 probe for all bacteria. The dashed lines indicate the boundary between the mucosal surface and the microbial biofilm. Regions showing direct contact between bacteria and the epithelium, or bacterial presence within the lamina propria, are marked with a star. Scale bars are 25 µm. (B) Biofilm damage scores are shown for each of the three experimental groups, with each dot representing the mean value for an individual mouse, calculated from 3–5 distinct microscopic fields (except 1 rat in TNBS + Dabigatran group). Statistical significance was determined by ANOVA followed by Dunnett’s test for multiple comparisons, where *P*<0.05 was considered significant.

As expected, the control group exhibited a dense microbial biofilm separated from the epithelial surface by a thick, sterile mucus layer (Figure 4A left panel). In contrast, the TNBS group showed significant biofilm disruption, with partial degradation of the mucus layer and bacterial infiltration into the submucosal tissues (Figure 4A, middle panel). Remarkably, local administration of the thrombin inhibitor Dabigatran completely reversed this phenotype, restoring the sterile mucus layer and preventing bacterial infiltration (Figure 4A, right panel). Moreover, quantitative assessment of microbiome biogeography confirmed a protective effect of thrombin inhibition on biofilm architecture (Figure 4B).^7^ These structural improvements may contribute to the previously reported anti-inflammatory effects of Dabigatran in this model (namely, reductions in myeloperoxidase activity, colon wall thickening, and macroscopic damage scores).^4^

These results identify thrombin as a major contributor to biofilm destabilization at the mucosal surface during colitis, highlighting its potential as a therapeutic target for preserving mucosal barrier integrity and mucosal biofilm architecture in CD.

### Thrombin-exposed biofilms have a pathogenic phenotype

**Figure 5.**
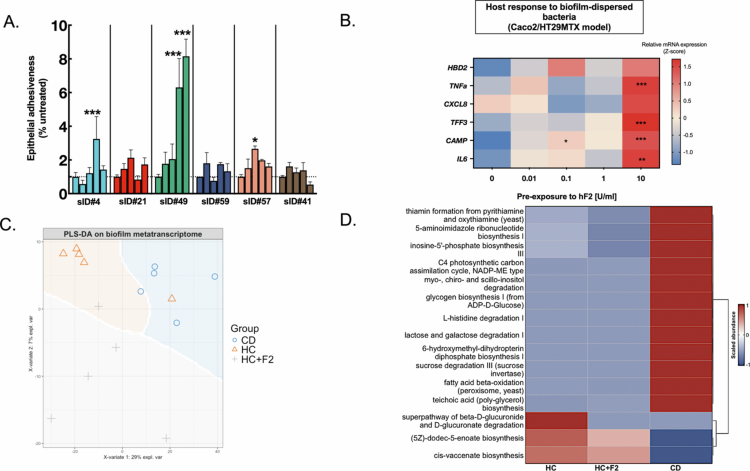
High thrombin-exposed biofilms have enhanced pathogenic properties and a distinct transcriptome profile. Mucosa-associated microbiota (isolated from colonic biopsies) from healthy individuals (n = 11) were cultured in vitro as a polymicrobial anaerobic biofilm and exposed to increasing concentrations of human purified thrombin (0, 0.01, 0.1, 1, and 10 U/mL). (A) Normalized counts of 6 biofilm-dispersed bacteria were cultured on the apical surface of the human Caco2/HT29MTX monolayer. After a 4-hour coculture, the number of bacteria that adhered to the epithelium was quantified. For each individual (subject ID or sID#), the raw values were normalized to the average values of the condition without thrombin (illustrated by the dotted line). The plot represents the mean values for all individuals in each condition (human purified thrombin concentrations ranging from 0 to 10 U/mL). Statistical significance was determined by 2way-ANOVA followed by Dunnett’s test for multiple comparisons, where P<0.05 was considered significant (* = *p* < 0.05, ** = *p* < 0.01, *** = *p* < 0.005). (B) After coculture with a normalized amount of biofilm-dispersed bacteria, epithelial cells were collected for RNA extraction. The heatmap represents the scaled relative expression of a targeted list of host response genes for each individual's and were grouped for each condition regarding thrombin exposure. Specifically, these genes are involved in innate inflammatory signaling (IL6, TNF, and IL8), secretion (HBD2), and antimicrobial responses (TFF3 and CAMP). The color gradient from blue to red corresponds to the scale of relative mRNA expression. Statistical significance was determined by ANOVA followed by Dunnett’s test for multiple comparisons, where *P*<0.05 was considered significant (* = *p* < 0.05, ** = *p* < 0.01, *** = *p* < 0.005). (C) Total RNA was sequenced using Illumina in cultured biofilms from either healthy controls (exposed or not to 10 U/mL purified human thrombin, *n*=5 in each group) or Crohn’s disease patients (*n*=5). Plot represents Partial Least Squares Discriminant Analysis (PLS-DA) of metacyc functional pathway profiles from meta-transcriptomic data of cultured biofilms, with background color representing class membership based on the Mahalanobis distance, aiding visualization of group separation in the first two PLS-DA components. (D) Heatmap is showing the top 15 most significantly differentially abundant metacyc functional pathways in cultured biofilms from the three groups (Kruskal‒Wallis test, *P* < 0.05). The rows represent pathways and the columns represent groups (*n*=5 HC, *n*=5 HC+F2, *n*=5 CD). The color gradient from blue to red indicates scaled abundance, with blue representing lower and red representing higher abundance levels.

Given that thrombin induces more unstable biofilms with increased bacterial dispersion, we next sought to assess whether bacteria dispersed by thrombin exposure exhibited increased pathogenic behavior on the surface of gut epithelium. To investigate this, we first exposed Caco-2 and HT29-MTX-E12 cell lines to bacteria dispersed from mature biofilms derived from healthy mucosa-associated microbiota that had been exposed for 24 H to human thrombin. We then compared the adhesion ability of these dispersed bacteria at different thrombin concentrations and found a significant increase at 10 U/mL (a 64% increase compared to unexposed biofilms; *p* = 0.0302) (Figure 5A). At the molecular level, we observed that bacteria from thrombin-exposed biofilms triggered a significantly enhanced epithelial immune response characterized by the upregulation of pro-inflammatory cytokines (qPCR: TNFα, IL-6) and antimicrobial effectors (CAMP, TFF3, HBD2) (Figure 5B).

To elucidate the mechanisms underlying the thrombin-induced pathogenic phenotype, we performed global metatranscriptomic profiling using short-read RNA sequencing on human biofilm microbiota. Each of the three groups of interest (healthy, healthy treated with thrombin, and CD) exhibited a distinct transcriptional signature (Figure 5C). Overall, the most discriminant shift was due to CD metatranscriptomes relative to both HC groups (Figure 5D, showing a scaled heatmap of the top 15 most statistically significant differentially abundant MetaCyc features). Further analysis revealed that a substantial portion of MetaCyc pathways and enzyme class (EC) were conserved across all conditions, for example, 63% of MetaCyc pathways were shared (Supplementary Figure 11A). However, several features were unique to individual groups, with, for instance, 15% of MetaCyc pathways and 26% of EC functions specific to CD samples (Supplementary Figure 12A). Multiple features were differentially abundant between CD and healthy control biofilms. For instance, CD biofilms showed increased expression of enolase (EC 4.2.1.11) and enrichment in the nucleotide metabolism superpathways (Supplementary Figures 11B, C). Despite the limited overlap between thrombin-treated healthy biofilms and those from CD patients, with only 2% shared metabolic features identified (e.g., the D-glucuronate metabolism superpathway; Figure 5D), thrombin exposure still induced distinct functional changes in healthy donor samples. These included enrichment of EC 2.7.1.30 (glycerokinase), EC 1.1.1.60 (NAD⁺/NADP⁺-dependent oxidoreductase), and alterations in the pantothenate and coenzyme A biosynthesis superpathways (Supplementary Figures 11 and 12).

Collectively, these findings indicate that elevated thrombin levels can drive a commensal microbiota toward a more pathogenic functional state, potentially mediated through specific transcriptomic alterations.

### Thrombin-exposed mucosa associated microbiota directly causes colon damage when transplanted into non-predisposed hosts

**Figure 6.**
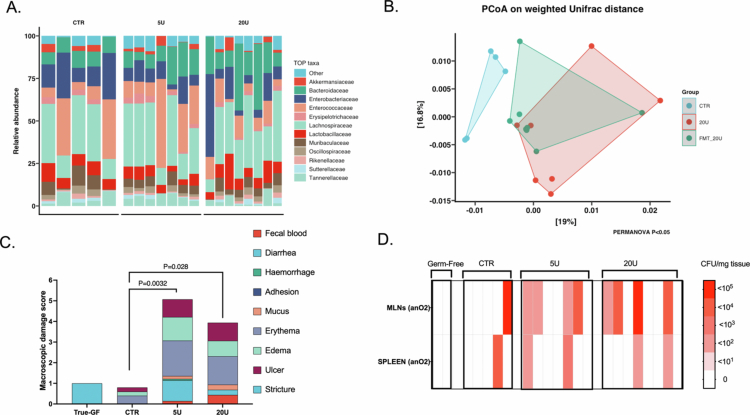
Thrombin-induced dysbiosis triggers macroscopic inflammation when transferred into non-predisposed mice. (A, B) C57Bl/6 mice (6–8 weeks old) were intracolonically exposed for 10 days to PBS (CTR, *n*=7), human purified thrombin at 5 UI/mL (5 UI/mL F2, *n*=7), and human purified thrombin at 20 UI/mL (20 UI/mL F2, *n*=8). (A) After 10 days of exposure, feces were collected for microbial taxonomy assessment using 16S rRNA metabarcoding. The stacked bar plots illustrate the relative abundance of the top 12 most abundant taxa at the Family rank. Each individual is depicted as a separate bar plot, highlighting taxonomic variations. (B) Beta-diversity (Bray–Curtis dissimilarity) of the fecal microbiota from each group was visualized using Principal Coordinate Analysis (PCoA) based on the weighted unifrac distance. PERMANOVA *P*-value >0.05 for all pairwise comparisons. (C, D) C57Bl/6 axenic mice (6‒8 weeks old) underwent oral gavage with the colonic mucosa-associated microbiota (fecal microbiota inoculation) from mice intracolonically exposed for 10 days to PBS via intrarectal injection (Ctrl, *n*=5), human purified thrombin at 5 UI/mL (5 U, *n*=7), human purified thrombin at 20 UI/mL (20 U, *n*=8). Two mice were not exposed before sacrifice (True-GF, *n*=2). (C) Four days after transplantation, the colons were harvested for macroscopic damage score. Statistical significance was determined by ANOVA followed by Dunnett's test for multiple comparisons, where *P*<0.05 was considered significant. (D). Four days after transplantation, the spleen and mesenteric lymph nodes (MLN) were collected and plated on Blood Agar to estimate bacterial translocation. The stacked bar plots illustrate the bacterial translocation as colony forming unit (CFU)/mg of tissue. Each individual is depicted as a separate bar plot, highlighting translocation variations.

We sought to evaluate whether thrombin could induce a pro-inflammatory behavior in healthy mucosa-associated microbiota *in vivo*. We first exposed C57BL/6 mice intracolonically to doses of active thrombin comparable to those detected in the mucosa of high-F2 CD patients. Mucosa-associated microbiotas were then isolated from the distal colon. Using 16S V3–V4 metabarcoding, we confirmed that thrombin exposure for 10 days (at concentrations of 5 and 20 U) did not cause a major shift in taxonomic composition (Figure 6A and B).

Next, we orally administered this thrombin-exposed mucosa-associated microbiota to germ-free C57BL/6 mice (10–14 weeks old). Colonized mice were sacrificed after 4 days to assess the host response to the thrombin-exposed microbiota. Following fecal microbiota transplantation, 16S profiling confirmed successful engraftment of the transplanted communities and revealed no significant taxonomic differences between the three experimental groups (Supplementary Figure 13). Macroscopic colon damage evaluation revealed significantly greater damage in recipient animals transplanted with thrombin-exposed microbiota (0.8 ± 1.3 vs 5.07 ± 1.59 in the 5 U group, *p* = 0.0032, Figure 6C), including an increased incidence of erythema, diarrhea, edema, and ulcers. Additionally, we observed increased bacterial translocation in the 5 and 20 U groups compared to the control group (Figure 6D).

We then analyzed the gene expression of several markers of the mucosal response of mice post-fecal microbiota transplantation. Specifically, we examined the relative expression of 92 genes involved in encoding pro-inflammatory cytokines, proteases, antimicrobials, immune system components, and tight junction proteins to identify patterns indicative of the host epithelial innate response to thrombin-exposed bacteria (multiplex microfluidic quantitative RT-PCR). The scaled expression heatmap revealed notable disparities between the thrombin-exposed (5 and 20 U) and unexposed groups (CTR; Supplementary Figure 14). A PCA ordination plot demonstrated a clear shift in the epithelial transcriptome of the mice exposed to thrombin-exposed bacteria compared to the control group (Supplementary Figure 15, PERMANOVA *P* < 0.05 for both pairwise comparisons between 5U/20U and CTR groups). Consistent with our previous work, we observed a significant upregulation of *F2r* (PAR-1) and *F2rl3* (PAR-4) genes dependent on thrombin exposure (indirectly through prior microbiota exposure; Supplementary Figure 16).^4^ Interestingly, we detected a significant defect in *Atg16l1* in both thrombin-exposed groups, a key pathway involved in autophagy, mirroring the defects observed in CD patients with the loss-of-function T300A mutation (Supplementary Figure 16).^16^ Conversely, we noticed an increase in *Lrf5* gene expression in the 5 U group, which has been associated with IBD disease activity and the regulation of Th1 and Th17 immune responses and cytokine production (Supplementary Figure 16).^17^ ^,^ ^18^

These results confirm that exposure of mucosa-associated microbiota to high thrombin levels, as observed in a subset of CD patients, can shift the microbiota toward a pathogenic state capable of inducing macroscopic inflammation.

### Thrombin-induced pathogenicity is linked to Toll-Like Receptor-5 (TLR5)/flagellin pathway modulation

An elevated systemic antibody response against bacterial flagellins has been suggested as a key driver of inflammation in CD.^19^ ^,^ ^20^ Since various flagellins genes are found in commensal metagenomes, even within the same core genome (e.g. *Lachnospiraceae*) and the levels of bioactive isoforms can vary according to environmental changes within the gut,^21^ ^,^ ^22^ we sought to assess the impact of thrombin on bioactive flagellins expression and release.

**Figure 7.**
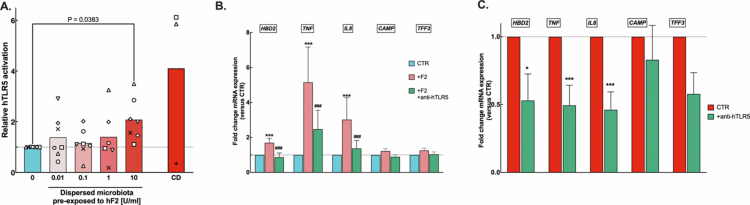
The inflammatory response induced by thrombin-associated dysbiosis is partially driven by the TLR5 pathway. Mucosa-associated microbiota (isolated from colonic biopsies) from up to 7 healthy individuals (distinct pictograms representing the microbiota of each individual) were cultured in vitro as a polymicrobial anaerobic biofilm and exposed to increasing concentrations of human purified thrombin (ranging from 0 to 10 U/mL). (A) Normalized counts of biofilm-dispersed bacteria were cultured on the apical surface of the human HEK-Blue™ hTLR5 monolayer. After a 4-hour coculture, some of the supernatant was collected and applied to QUANTI-Blue medium. TLR5 activity was measured with a spectrophotometric assay after overnight exposure. For each individual, the raw values were normalized to the average values of the condition without thrombin. The plot represents the mean values for all individuals in each condition (human purified thrombin concentrations ranging from 0 to 10 U/mL). Statistical significance was determined by ANOVA followed by Dunnett’s test for multiple comparisons, where *P*<0.05 was considered significant. (B) Normalized counts of biofilm-dispersed bacteria from colonic biopsies of 11 healthy subjects were cultured on the apical surface of the human Caco2/HT29 monolayer. Four conditions were performed: unexposed cells, cells exposed to bacteria dispersed from native biofilms (0 UI F2), cells exposed to bacteria dispersed from thrombin-exposed biofilms at 10 UI/mL (10 UI F2) and cells treated 1 h before interaction with 1.0 µg/mL of neutralizing anti-TLR5 antibody and then exposed to bacteria dispersed from thrombin-exposed biofilms at 10 UI/mL (10 UI F2 + Ab TLR5). After 4 h of coculture, epithelial cells were collected for RNA extraction. A scatter plot depicts the relative mRNA expression of a targeted list of host response genes for each individual. Specifically, these genes are involved in innate inflammatory signaling and secretory responses (BD2). (C) Normalized counts of biofilm-dispersed bacteria from the stools of 3 Crohn’s disease patients were similarly cultured on the apical surface of the human Caco2/HT29 monolayer with and without pre-exposure to 1.0 µg/mL of neutralizing anti-TLR5 antibody. Statistical significance was determined by ANOVA followed by Dunnett’s test for multiple comparisons, where P<0.05 was considered significant (* = *p* < 0.05, ** = *p* < 0.01, *** = *p* < 0.005).

To investigate this, we first exposed a human HEK-Blue™ hTLR5 cell line to dispersed bacteria derived from mature biofilms, which were cultured from mucosa-associated microbiota and then exposed to human thrombin. We compared the ability of these dispersed bacteria to trigger the TLR5 pathway at different thrombin concentrations and found a significant increase at 10 U/mL (a 59.7% increase compared to unexposed biofilms; *p* = 0.0375) (Figure 7A). We cocultured Caco-2 and HT29-MTX-E12 cell lines with dispersed bacteria previously exposed to high thrombin levels (10 U/mL), after antagonizing these cells with an anti-TLR5 antibody. TLR5 pathway antagonization significantly reversed the inflammatory (qPCR: *TNFα, IL-8*) and antimicrobial epithelial response (*HBD2*) induced by microbiota thrombin exposure (Figure 7B). Similarly, TLR5 pathway antagonization significantly reversed the inflammatory (qPCR: *TNFα, IL-8*) and antimicrobial epithelial response (*HBD2*) induced by dispersed bacteria from biofilms cultured from CD fecal microbiota patients (Figure 7C). Therefore, altogether these results confirm a role for the TLR5 pathway both in CD pathogenesis and in the host response to microbiota that have been exposed to Crohn's associated doses of mucosal thrombin.

To explore the mechanism behind thrombin-mediated TLR5 modulation, we examined whether thrombin directly cleaves flagellins into more immunostimulatory fragments. First, we confirmed that supernatants from thrombin-exposed biofilms contained higher levels of TLR5-activating flagellins (Supplementary Figure 17A). However, further exposure of these supernatants to thrombin did not produce cleaved flagellin fragments capable of enhancing TLR5 activation, suggesting no direct proteolytic effect of thrombin on flagellins (Supplementary Figure 17B). These findings imply that thrombin does not post-translationally modify flagellins but may instead modulate their expression by the microbiota. Finally, we investigated whether adherent-invasive *Escherichia coli* (AIEC), a well-characterized biofilm-forming pathobiont implicated in CD, could be a target of thrombin-induced virulence mechanisms.^20^ We examined several *AIEC* strains, including the reference strain LF82 wild-type, its isogenic ΔfliC mutant (deficient in flagellin, to assess the contribution of flagellin-dependent pathways), and the NRG strain. Across all conditions, thrombin exposure did not induce any significant changes in biofilm biomass or dispersal (Supplementary Figure 18A and B). In addition, three clinical *E. coli*/*Enterobacteriaceae* isolates obtained from patient-derived mucosal microbiota were tested, but none exhibited enhanced TLR5 activation upon thrombin treatment (Supplementary Figure 18C).

Altogether, these results suggest that thrombin-induced pathogenicity is partially mediated through the modulation of flagellin expression and the TLR5 signaling pathway, but does not appear to involve the commensal *Enterobacteriaceae* as a key effector.

## Discussion

Gut dysbiosis is a fundamental aspect of CD pathogenesis, marked by the depletion of beneficial microbial species, the proliferation of pathobionts, and an overall reduction in microbial diversity.^23^ While numerous studies have focused on the role of the immune system and antimicrobial peptides in shaping the intestinal microbiota, far less attention has been given to non-immune epithelial factors, despite their direct and continuous interaction with the luminal microbial community.^24^ Understanding these epithelial-derived signals is critical to elucidate the mechanisms that drive microbial imbalance in CD and to identify new biomarkers and therapeutic targets.^25^

Among the potential epithelial regulators, proteases represent compelling but underexplored candidates. These enzymes are abundantly secreted at the mucosal surface and can exert direct effects on microbial behavior, biofilm organization, and host–microbe interactions.^26^ ^,^ ^27^ Recent studies have pointed to proteolytic dysregulation as a key feature of CD, with several proteases (most notably thrombin) being found to be upregulated in the intestinal mucosa of patients with active and inactive disease.^3^ ^,^ ^4^ ^,^ ^28^ ^,^ ^29^ Beyond its classical functions, thrombin influences the spatial architecture of mucosa-associated biofilms in physiological conditions, preventing bacterial translocation across the intestinal epithelium.^7^ ^,^ ^30^ Therefore, drastic dysregulation of thrombin expression and activity, such as that observed in a subset of CD patients is meant to strongly affect the microbiome. Indeed, here we demonstrated that CD-associated thrombin activity induces significant structural and metabolic changes in colonic biofilms, promoting the emergence of pathobionts capable of triggering mucosal inflammation, while not causing major shifts in the overall taxonomic composition.

Our study emphasizes the need to go beyond taxonomic considerations and to assess the mucosa-associated microbiota in its natural state as a complex community embedded in a host- and self-produced matrix known as a biofilm.^27^ Primarily viewed from a microbiology perspective, the concept of biofilms has now gained recognition as a clinically and disease-relevant phenomenon.^31^ The results present herein indicate that high levels of thrombin exposure, as seen in some CD patients, destabilize biofilms, leading to an increased dispersal rate and a concurrent decrease in biofilm mass. These physical changes in biofilms have previously been identified as early steps in the onset of virulence.^15^ ^,^ ^30^ ^,^ ^32^ Importantly, these findings underscore the limitations of taxonomic analyses alone in capturing the full scope of dysbiosis. The traditional view of dysbiosis as a mere imbalance in microbial composition must be expanded to include functional, transcriptomic, and architectural dimensions. The spatial organization and cohesion of the mucosa-associated microbiota is increasingly recognized as a key feature of intestinal homeostasis.^27^ Loss of this structure, as induced by thrombin, can promote closer and denser microbial‒epithelial contacts, immune activation, and mucosal barrier disruption, even in the absence of major taxonomic shifts. Importantly, we observed here that thrombin inhibition with dabigatran can mitigate the biofilm disorganization associated with colitis in a rat model, providing the first evidence of thrombin inhibition as a potential therapeutic approach as a potential therapeutic approach aimed at maintaining biofilm homeostasis.

Using *in vitro* approaches, we demonstrated that bacteria dispersed from thrombin-exposed biofilms exhibit enhanced pathogenic properties, including increased adhesion to the epithelium and a greater capacity to induce inflammation and antimicrobial responses. Recognizing that the interaction between the gut microbiome and its host is bidirectional and more complex than what our model can fully capture, we employed a fecal microbiota transfer approach to elucidate the role of thrombin-induced dysbiosis, potentially related to the ability of thrombin to induce colitis.^4^ This experiment clearly demonstrated that prior exposure of the mucosa-associated microbiota to thrombin is sufficient to induce a virulent phenotype that causes macroscopic inflammation when transplanted into a non-predisposed host. At a molecular level, thrombin-exposed microbiota activates a number of host defense mechanisms. Notably, thrombin-exposed microbiota impaired autophagy, as evidenced by decreased ATG16L1 RNA levels, potentially facilitating pathobiont colonization and increasing pathogenicity. Previous studies have sought to understand the interplay between autophagy and the microbiota in the context of IBD.^8^ For example, AIEC has been shown to inhibit ATG16L1 expression through the upregulation of Mir30c and Mir130a in intestinal epithelial cells.^33^ Thrombin-exposed microbiota also increased the expression of protease signal receptors (PAR1 and PAR4), known to affect barrier functions and to contribute to the host inflammatory response, thus possibly maintaining the proteolytic imbalance in a vicious cycle.^4^ ^,^ ^34^

We identified the modulation of flagellins as a mechanism underlying the changes in mucosa-associated microbiota induced by thrombin in CD. Similar to what has been previously shown with AIEC,^20^ we observed in complex microbiota samples that flagellins/TLR5 signaling is a critical downstream pathway modulated by thrombin. Interestingly, thrombin’s effect is not attributable to direct cleavage or post-translational modification of flagellins, but rather to its indirect transcriptional regulation of bacterial genes. This may explain the sustained anti-flagellins immune response observed in some CD patients, potentially linking proteolytic imbalance with antigen exposure and immune activation.^35^ Although our results on AIEC strains were inconclusive, the contribution of *Enterobacteriaceae* cannot be entirely excluded, as this family comprises highly heterogeneous strains in the context of CD, which may not be fully represented by the LF82 and NRG strains used here. This limitation is consistent with our observation that thrombin does not uniformly affect biofilms formed by different individual bacterial strains *ex vivo.* ^7^ We propose that thrombin may induce community-level functional alterations (including changes in biofilm structure, microbial spatial organization, and interspecies interactions) that increase the exposure, accessibility, or functional impact of pre-existing flagellin at the mucosal interface, thereby enhancing TLR5 signaling without requiring transcriptional upregulation and leading to greater antigen exposure. While Zhao et al. found that elevated serum IgG specific to *Lachnospiraceae* was linked to disease complications, we could not identify correlations between thrombin levels and disease activity, suggesting that thrombin-associated microbial functional changes represent a distinct dimension of the host‒microbiota interaction not fully captured by standard clinical or endoscopic activity scores.

Interestingly, while thrombin did not induce global taxonomic changes, it reshaped microbial functionality, as revealed by metatranscriptomic and metagenomic analysis. A significant enrichment of the pantothenate and Coenzyme A biosynthesis pathway was observed – an axis known to regulate virulence genes and secretion systems (notably T3SS) in enteropathogens.^36^ Interestingly, previous work on *Salmonella* has revealed the coregulation of T3SS and flagellar genes.^37^ This finding suggests that thrombin does not simply alter who is present in the microbiota, but rather what they do, reinforcing the importance of functional over compositional dysbiosis.

These findings should be interpreted in light of several limitations. First, working with a complex polymicrobial community, while pathophysiologically relevant, prevented precise identification of the key bacterial strains implicated in thrombin-induced pathogenicity, an inherent challenge given that the impact of individual strains on host–microbiota interactions varies considerably depending on interspecies context.^38^ ^,^ ^39^ Second, all analyses are based on single-timepoint measurements, which precludes inference about longitudinal dynamics. Elevated thrombin levels observed in patients in clinical or endoscopic remission may reflect either a pre-clinical signal of impending relapse or a post-flare decreasing phase, distinctions that only longitudinal sampling across disease phases could resolve. Third, a major open question concerns the upstream mechanisms responsible for the elevated epithelial thrombin activity observed in a subset of CD patients. Analysis of clinical and biological profiles did not delineate a clearly homogeneous subgroup, likely reflecting both the limited cohort size and the strong inter- and intra-individual variability of thrombin expression over the disease course. Thrombin hyperactivity may therefore not represent a universal signature of CD, but rather a specific disease subphenotype characterized by disrupted host–microbiota dialogue at the mucosal surface. Identifying this subgroup will be a key step toward personalized therapeutic strategies. Finally, the use of *ex vivo* and reductionist biofilm models may not fully recapitulate *in vivo* conditions; current functional annotation tools may lack resolution for specific microbial processes such as flagellin regulation; and our hypothesis-driven approach inevitably captures only a fraction of CD complexity, leaving open the possibility that alternative or parallel pathways contribute to microbial dysfunction and pathogenicity. In addition, while our data suggest increased bacterial translocation and altered epithelial organization, whether thrombin-driven microbial changes directly impair epithelial barrier permeability remains to be functionally established. Taken together, these limitations underscore the need for longitudinal, high-resolution, and mechanistically integrated studies to fully elucidate the role of thrombin in CD pathogenesis.

## Conclusion

These findings shed light on a novel mechanism linking proteolytic imbalance and microbial pathogenicity at the mucosal surface in CD. We identify thrombin not only as a factor involved in the pathophysiology of CD but also as a host functional effector capable of reshaping mucosa-associated biofilms, enhancing microbial pathogenicity, and triggering host inflammation, even in non-predisposed individuals. This study emphasizes the need to move beyond taxonomy in microbiome research and to integrate functional and structural assessments of the microbial ecosystem. By demonstrating that thrombin-exposed microbiota alone are sufficient to initiate inflammation, these results raise the possibility that targeting host–microbiota interactions, particularly those mediated by proteases, may offer new therapeutic avenues in the subset of CD patients in whom this pathway is particularly prominent. Ultimately, this work positions thrombin as both a driver and amplifier of gut dysbiosis and inflammation, as well as a potential biomarker of microbial biofilm dysfunction and pathogenicity.

## Supplementary Material

Supplementary Material Supplementary data

REVISED_suppFigures_ADOBE.pdf REVISED_suppFigures_ADOBE.pdf

## Data Availability

The 16S rRNA sequences, metagenomic and metatranscriptomic data, and other biological information have been deposited in public NCBI Sequence Read Archive (SRA) databases under the accession ID: PRJNA1279062. Additional biological information or raw data can be obtained upon reasonable request through the corresponding author.

## Abbreviations

BMI: Body mass index
CD: Crohn’s disease
CRP: C-reactive protein
EC: Enzyme class
FISH: Fluorescent *In Situ* hybridization
HC: Healthy control
IBD: Inflammatory bowel disease
PARs: Protease-activated receptors
PBS: Phosphate buffered saline
TLR5: Toll-Like Receptor-5
TNBS: Trinitro benzene sulfonic acid
UC: Ulcerative colitis
