# Supplementary material for: High thrombin activity associated with crohn’s disease induces microbiota pathogenicity contributing to mucosal inflammation
Source: Gut Microbes. 2026 Jun 17;18(1):2687903. doi: 10.1080/19490976.2026.2687903 (PMC13285544; doi:10.1080/19490976.2026.2687903)
Supplement: Supplementary Material — Supplementary data [file KGMI_A_2687903_SM9107.docx]

**Supplementary data**

**Supplementary Table 1. Healthy human cohort characteristics**

|  | Healthy individuals  (n=11) |
| --- | --- |
| Gender |  |
| Male | 10 (90.9) |
| Female | 1 (9.1) |
| Mean age (years) | 58 (± 9.8) |
| Mean Charlson Comorbidity Index | 1.8 (± 1.5) |
| Smoking Status  Active  Past  Never smoker | 2 (18.2)  2 (18.2)  7 (63.6) |
| BMI | 26.7 (± 2.5) |
| Familial history of Inflammatory Bowel Disease | 0 (0) |

Data are represented as mean (± standard deviation) for continuous variables and n (%) for categorical variables.

BMI: Body Mass Index

**Supplementary Table 2. Demographic and Clinical Characteristics of Patients Providing Stool Samples**

|  | Healthy individuals  (n=9) | Crohn’s disease  (n=37) | Ulcerative Colitis (n=9) |
| --- | --- | --- | --- |
| Gender  Male  Female | 4 (44.4)  5 (55.6) | 15 (40.5)  22 (59.5) | 2 (22.2)  7 (77.8) |
| Mean age (years) | 36.3 (± 14.3) | 41.9 (± 15.3) | 42.6 (±14.4) |
| Age at diagnosis*^†^*  A1  A2  A3 |  | 2 (5.4)  28 (75.7)  7 (18.9) | 0 (0)  8 (88.9)  1 (11.1) |
| Crohn’s disease  Location*^†^*  L1  L2  L3  L4  Behavior *^†^*  B1  B2  B3  Perineal disease  Harvey Bradshaw Index |  | 13 (35.1)  10 (27)  11 (29.8)  3 (8.1)  23 (62.2)  9 (24.3)  5 (13.5)  7 (20.6)  2.7 (± 3.6) |  |
| Ulcerative colitis  Extension*^†^*  E1  E2  E3  Mayo clinical subscore |  |  | 3 (33.3)  5 (55.5)  1 (11.1)  1.1 (± 2.1) |
| Extra intestinal manifestations |  | 4 (10.8) | 3 (33.3) |
| History of bowel resection |  | 12 (23.4) | 0 (0) |
| Ongoing IBD treatments  5 amino salicylates  Thiopurines  Methotrexate  Anti TNFs  Ustekinumab  Vedolizumab  JAK inhibitors |  | 3 (8.1)  9 (24.3)  1 (2.7)  13 (35.1)  4 (10.8)  5 (13.5)  1 (2.7) | 4 (44.4)  1 (11.1)  4 (44.4)  4 (44.4)  0 (0)  2 (22.2)  0 (0) |

Data are represented as mean (± standard deviation) for continuous variables and n (%) for categorical variables.

†: According to the Montreal classification

**Supplementary Table 3. Demographic and Clinical Characteristics of Patients Providing Colonic Biopsies for thrombin activity assessment**

|  | Healthy individuals  (n=8) | Crohn’s disease  (n=5) |
| --- | --- | --- |
| Gender  Male  Female | 2 (40)  6 (60) | 2 (40)  3 (60) |
| Mean age (years) | 59.6 (± 10.4) | 41.8 (± 11.9) |
| Age at diagnosis*^†^*  A1  A2  A3 |  | 1 (20)  4 (80)  0 (0) |
| Crohn’s disease  Location*^†^*  L1  L2  L3  L4  Behavior *^†^*  B1  B2  B3  Perineal disease |  | 3 (60)  1 (20)  1 (20)  0 (0)  2 (40)  1 (20)  2 (40)  2 (40) |
| Extra intestinal manifestations |  | 2 (40) |
| History of bowel resection  Rutgeerts score  i0  i1  i2  i3  i4 |  | 3 (60)  0 (0)  0 (0)  1 (33.3)  1 (33.3)  1 (33.3) |
| Mean CDEIS score (among non-operated patients) |  | 0 (± 0) |
| Ongoing IBD treatments  5 amino salicylates  Thiopurines  Methotrexate  Anti TNFs  Ustekinumab  Vedolizumab  JAK inhibitors |  | 1 (20)  1 (20)  0 (0)  3 (60)  1 (20)  0 (0)  0 (0) |

Data are represented as mean (± standard deviation) for continuous variables and n (%) for categorical variables.

†: According to the Montreal classification

**Supplementary Table 4.** List of qRT-PCR human primers used in the study

| **Gene** | **Forward 5’- 3’** | **Reverse 5’- 3’** |
| --- | --- | --- |
| CAMP | TGTGCTTCGTGCTATAGATGG | TGAAGTCACAATCCTCTGGTG |
| IL6 | TCAATATTAGAGTCTCAACCCCCA | GAAGGCGCTTGTGGAGAAGG |
| IL8 | GCCTTCCTGATTTCTGCAGCT | TGCACTGACATCTAAGTTCTTTAGCAC |
| TNF | ACTTTGGAGTGATCGGCC | GCTTGAGGGTTTGCTACAAC |
| BD2 | CCATGAGGGTCTTGTATCTCC | AGGGCAAAAGACTGGATGAC |
| HPRT | CCTGGCGTCGTGATTAGTGA | CGAGCAAGACGTTCAGTCCT |
| GAPDH | GAGAAGGCTGGGGCTCAT | TGCTGATGATCTTGAGGCTG |
| TFF3 | GCTCTGCTGAGGAGTACGTG | GGGATCCTGGAGTCAAAGCA |

**Supplementary Table 5.** List of qRT-PCR mice primers used in the study

| **Gene** | **Forward 5’- 3’** | **Reverse 5’- 3’** |
| --- | --- | --- |
| m_PAR-1 | TCTTCCCGCGTCCCTATGA | GGGGTTCACCGTAGCATCTG |
| m_PAR-2 | GGACCGAGAACCTTGCAC | GAACCCCTTTCCCAGTGATT |
| m_PAR-3 | AGCTGAGGGGAATCTACGCT | AGGTTGGCTTTGCTGAGTTG |
| m_PAR-4 | TGCGTAGACCCTTTCATC | GGGTTCAAGAGGGATGTAG |
| m_SERPIN A1 | CAACAATGGGGCTGACCTCT | CGATGGTCAGCACAGCCTTA |
| m_SERPIN E2 | TGTGGAAGTCTCGGTTTC | GGTAGTGATGTGAGGGATG |
| m_ADAM10 | GAAGATGGTGTTGCCGACAG | TTTCCATACTGACCTCCCAGC |
| m_MMP7 | GCAGGCATTCAGAAGTTATATG | ACAAGGAAGAGGGAAACAG |
| m_TIMP1 | GAGCCCTGCTCAGCAAAGAG | GGACCTGATCCGTCCACAAAC |
| m_TIMP2 | CAACAGGCGTTTTGCAATGC | ATCCTCTTGATGGGGTTGCC |
| m_TFPI | AACATCGTGGTTCCCCAGTC | GCTTAGGCGCTCCTTTCTCA |
| m_F2 | AACCTGCCCATTGTAGAGCG | CAGCGGTTGTTAAAGGGGCT |
| m_F5 | GGCATGCAAACGCCATTTCT | TCTGGGCTCCCAATAAGTCAG |
| m_F10 | GGAACGTGCCAACAATGTCC | CACACTGGTCGCCGTCTTTA |
| m_PRSS2 | GCTTTCCCTGTGGATGATGA | AGTGGTAGCCAGCATTTAGG |
| m_PIGR | CTATTGGTGTCTTACCAATGGTGACT | CTGTTGCGTTCTGTGGCGT |
| m_CLDN1 | CCTACTTTCCTGCTCCTG | TGTCCATTTTGTATTTGCTCC |
| m_CLDN2 | CCCACAGATACTTGTAAGGAG | CCAAAAGGCCTAGGATGTAG |
| m_CLDN3 | ACTGCGTACAAGACGAGACG | GGGCACCAACGGGTTATAGA |
| m_CLDN4 | CCACTCTGTCCACATTGCCT | CTTTGCACAGTCCGGGTTTG |
| m_CLDN5 | CAATGGCGATTACGACAAG | GGC-TAG-TGA-TGG-TCA-ACG |
| m_CLDN7 | ACGCCCATGAACGTTAAGTACGAG | CTTTGCTTTCACTGCCTGGACA |
| m_OCCLUDIN | ACCCTGACCACTATGAAAC | CGTCTAGTTCTGCCTGTAAG |
| m_MUC2 | GTAAACTGCTCTCTGGACTG | CTTGGAAGACGTGGTAGATG |
| m_MUC5AC | GCATCTCCTACTGCCAAG | TGTGTCCCCTAAAGCATG |
| m_ZO1 | CGTTATGATCCAGCCCAG | GCTGGTTTACTCTGAGATGG |
| m_IL6 | CGTGGTTGTCACCAGCATCA | CTCTGCAAGAGACTTCCATCCAGT |
| m_IL10 | ATCGATTTCTCCCCTGTGAA | TGGCCTTGTAGACACCTTGG |
| m_IL15 | CATCCATCTCGTGCTACTTGTG | GCCTCTGTTTTAGGGAGACCT |
| m_IL22 | ACCGCTGATGTGACAGGAGC | AGGTGGTGCCTTTCCTGACC |
| m_IL25 | AACAGCAGGGCCATCTCTC | ACCCGATTCAAGTCCCTGTC |
| m_IL33 | GCTGCGTCTGTTGACACATT | CACCTGGTCTTGCTCTTGGT |
| m_IL36a | CAGCATCACCTTCGCTTAGAC | AGTGTCCAGATATTGGCATGG |
| m_IL36b | GCCTCATTGCCTGCTGTCATA | AAGCCATCATTGCCTTGGTC |
| m_IL36G | ACCACACCCGGACAGGTGGA | TGGGGTTGCCAGTCTTGGAGGA |
| m_IL1beta | ACCTTCCAGGATGAGGACATGAG | CATCCCATGAGTCACAGAGGATG |
| m_IFNgamma | GCGTCATTGAATCACACCTG | TGAGCTCATTGAATGCTTGG |
| m_TFF3 | CCTGGTTGCTGGGTCCTCTG | GCCACGGTTGTTACACTGCTC |
| m_TNFrF19 | TGGGAAGACAGGGAAAAC | GGCTGAAAGGATGGAAATG |
| m_TGFbeta | GACCCCCACTGATACGCCT | GCTGAATCGAAAGCCCTGTA |
| m_TNFalpha | GGTCCCCAAAGGGATGAG | AGGGTCTGGGCCATAGAACT |
| m_S100G | TCACCTGCTGTTCCTGTCTGA | GCCTTCCTTGGCTGCATATTT |
| m_MIF | CGGACCGGGTCTACATCAAC | GCATCGCTACCGGTGGATAA |
| m_COX2 | TGAGTCATTCACCAGACAG | GCAGCCATTTCCTTCTCT |
| m_F4/80 | AAGGACACGAGGTTGCTGACC | GCCAATCTGGAAAATGCCC |
| m_LY6G | TGCTCATCCTTCTTGTGGTCC | ATTGTAGCACTCCAGCCCCTG |
| m_CXCL1 | AGCCACACTCAAGAATGGTC | GTCAGAAGCCAGCGTTCAC |
| m_TLR-9 | CCAGACGCTCTTCGAGAACC | GTTATAGAAGTGGCGGTTGT |
| m_CCL2 | TTCTTTGGGACACCTGCTG | TGTTGGCTCAGCCAGATGCA |
| m_CD74 | CAAGTGCGACGAGAACGGTA | CAGGGTGACTTGACCCAGTTC |
| m_CSF1 | GCCAAGGAGGTGTCAGAACA | AGCATTGGGGGTGTTGTCTT |
| m_INOS | GAGATTGGAGTTCGAGACTTCTGTG | TGGCTAGTGCTTCAGACTTC |
| m_SLPI | TTACCTTTCACGGTGCTCCTTG | CTCCCAGTCAGTACGGCATTG |
| m_TLR2 | CAGCTTAAAGGGCGGGTCAGAG | TGGAGACGCCAGCTCTGGCTCA |
| m_TLR4 | GACACCAGGAAGCTTGAATCC | TCTGATCCATGCATTGGTAGG |
| m_REG3G | CGACACTGGGCTATGAAC | TCTCCACTTCAGAAATCCTG |
| m_CAMP | AAGGAACAGGGGGTGGTG | CCGGGAAATTTTCTTGAACC |
| m_DEFB6 | GTCTCCACTTGCAGCCTTTTC | AAACCTCCATTGCATGAACGC |
| m_BD-1 | CTGGCTGCCACCACTATGAAA | TTGTGAGAATGCCAACACCTGG |
| m_BD-2 | GCCATGAGGACTCTCTGCTC | AGGGGTTCTTCTCTGGGAAA |
| m_TRPV1 | CGGAAGACAGATAGCCTGAA | GCTCCATTCTCCACCAAGAG |
| m_TRPV4 | TCTTCACCCTCACCGCCTACTA | TCCACTGTGGTCCGGTAAGG |
| m_ACHE | CCTGGGTTTGAGGGTACTGA | GGTTCCCACTCGGTAGTTCA |
| m_CHRM1 | AGCAGCAGCTCAGAGAGGTC | GCCTGTGCCTCAGGATCTAC |
| m_CHRM2 | GATCCCACTTGGAAATCGCC | TTCTCCCTGGATCTGGCTTTC |
| m_CHRM3 | TGGTGTGTTCTTCCTTGGAC | ACCCAGGAAGAGCTGATGTT |
| m_HRH1 | TCACTCCAGGCCTCACATG | CAAAGTTCTCATCCCAAGTTTCCA |
| m_TRPA1 | GCTGGTGATGGATGAAGACA | ATGGACACATTGAAGCCAAG |
| m_BCHE | GGGCAGTAAAGCATCCTGAG | GAGGGGAGAACGAACCTTTC |
| m_TRPM8 | AGCTCTTGGCTGATTGAGCA | CCAAATCACTGTCACTGTAGGA |
| m_HTR1A | TACTCCACTTTCGGCGCTTT | GGCTGACCATTCAGGCTCTT |
| m_PENK | CGACATCAATTTCCTGGCGT | AGATCCTTGCAGGTCTCCCA |
| m_CGRP1 | CCAGGTGAGCCCTAAAATTCCT | TGCTTTCCAAGATTGACCTCAAAG |
| m_CD24a | GGCACTGCTCCTACCCACGC | CACCCCCTCTGGTGGTAGCG |
| m_CD44 | TCTGCCATCTAGCACTAAGAGC | GTCTGGGTATTGAAAGGTGTAGC |
| m_GSK3B | TGGTGCTGGACTATGTTC | GTTCTGTGGTTTAATGTCTCG |
| m_IRF5 | CCCCCACATGTTGCCTTTG | GTCCCCGGTACTGGAACTTGA |
| m_CDHE | TGACTCGAAATGATGTGGCTCCCA | ACTGCCCTCGTAATCGAACACCAA |
| m_ANXA2 | AAGGGAGGCTCTCAGCGATA | TGACCTCATCCACTCCTTTGG |
| m_CHGA | TCCCCACTGCAGCATCCAGTTC | CCTTCAGACGGCAGAGCTTCGG |
| m_EGF | AAGGATCCTGACCCCGAACT | TGGGGCATGTGCAGTGATAG |
| m_HNF1 | AAAACCCCAGCAAGGAAGAG | GGTTGGCAAACCAGTTGTAG |
| m_KLF4 | GACTAACCGTTGGCGTGAGG | GTCTAGGTCCAGGAGGTCGT |
| m_MAPK3 | CACTGGCTTTCTGACGGAGT | GGATTTGGTGTAGCCCTTGGA |
| m_BMI1 | TCCCCACTTAATGTGTGTCCT | CTTGCTGGTCTCCAAGTAACG |
| m_ATF3 | TTCTCTGACTCTTTCTGCAGGC | GCTGCCAAGTGTCGAAACAA |
| m_ATF4 | GCTGTCTGCCGGTTTAAGTTG | TGGATTTCGTGAAGAGCGCCAT |
| m_ATF6 | TCAGCTGATGGCTGTCCAGT | AGAAGCCTTGGTAACTTCCAGG |
| m_ATG16L1 | CCGAATCTCCCCTTTTGGGA | CATGCGCATCGAAGACATACG |
| m_XBP1s | GCTGAGTCCGCAGCAGGT | CAGGGTCCAACTTGTCCAGAAT |
| m_XBP1t | TGAAAAACAGAGTAGCAGCGCAGA | CCCAAGCGTGTTCTTAACTC |
| m_XBP1u | CAGACTACGTGCACCTCTGC | CAGGGTCCAACTTGTCCAGAAT |
| m_GAPDH | AGGTCGGTGTGAACGGATTTG | TGTAGACCATGTAGTTGAGGTCA |
| m_HPRT | TCAGTCAACGGGGGACATAAA | GGGGCTGTACTGCTTAACCAG |
| m_TBP | CAGCCTTCCACCTTATGCTC | TTGCTGCTGCTGTCTTTGTT |

**Supplementary Figure 1: Study design and experimental workflow.** Stool samples (1) were collected from healthy controls (HC, n = 9), Crohn’s disease (CD) patients (n = 37), and ulcerative colitis (UC) patients (n = 9) for thrombin (F2) quantification by Western blot, microbiota taxonomic profiling using 16S rRNA gene sequencing, and functional prediction using PICRUSt2. Colonic biopsies (2) were obtained from HC (n = 8) and CD patients (n = 5) for thrombin activity quantification. Additional biopsies from HC (n = 11) were used for microbiota taxonomic analysis, shotgun metatranscriptomics, ex vivo biofilm formation, and phenotyping assays, including co-culture with intestinal epithelial cell lines. TNBS-induced colitis was performed in male Wistar rats (3) with or without dabigatran (a thrombin inhibitor) to assess colonic damage. A thrombin-induced colitis model was established in C57BL/6 mice (4) to assess colonic damage and microbiota alterations. Germ-free C57BL/6 mice (5) were inoculated with colonic associated microbiota exposed to thrombin to investigate the impact on host colonic damage. Animal colonic damage was assessed by macroscopic scoring and fluorescence in situ hybridization (FISH), with additional qPCR analysis where indicated.

**Supplementary Figure 2: Thrombin western blot analysis in human feces.** Thrombin over-expression was confirmed by Western blot analysis using anti-thrombin antibody on stool samples from healthy controls (HC, n = 9), Crohn’s disease (CD) patients (n = 37), and ulcerative colitis (UC) patients (n = 9). Representative blots from 4 independent experiments are shown (2-3 patients per group).

**Supplementary Figure 3: Taxonomic analysis of feces from healthy donors and Crohn's disease (CD) patients (Grouped abundance).** Microbiota from fecal samples were precultured overnight in BHI under anaerobic conditions and DNA was extracted to seek for taxonomic differences between healthy individuals (n=4), high (n=8) and low thrombin individuals with CD (n=8). The bar plots illustrate the relative abundance of the TOP15 taxa. Individuals are depicted as a summary bar plot for each condition corresponding to the mean of the relative abundance for each individual.

**Supplementary Figure 4: Taxonomic analysis of feces from healthy donors and Crohn's disease (CD) patients (Alpha and Beta diversity).** Microbiota from fecal samples were precultured overnight in BHI under anaerobic conditions and DNA was extracted to seek for taxonomic differences between healthy individuals (n=4), high (n=8) and low thrombin individuals with CD (n=8). **(A)** The boxplots display values of alpha-diversity indices calculated for each individual within the study groups (Observed, Chao1, Shannon and Fisher). Boxes represent the interquartile range between the 25th and 75^th^ percentiles, and the horizontal line inside the box defines the median. Statistical significance was determined by ANOVA followed by Tukey’s test for pairwise comparisons, where P<0.05 was considered significant (* = p < 0.05, ** = p < 0.01, *** = p < 0.005). **(B)** Overall diversity dissimilarity of fecal microbiota from each group was visualized using Principal Coordinate Analysis (PCoA) based on the weighted unifrac distance. PERMANOVA P-value >0.05 for all pairwise comparisons**.**

**Supplementary Figure 5: Taxonomic analysis of feces from healthy donors and Crohn's disease (CD) patients (LEfSe).** Microbiota from fecal samples were precultured overnight in BHI under anaerobic conditions and DNA was extracted to seek for taxonomic differences between healthy individuals (n=4), high (n=8) and low thrombin individuals with CD (n=8). Multigroup differential taxa abundance was performed using Linear discriminant analysis effect size (LEfSe) and linear discriminant analysis (LDA) based on amplicon sequence variants is presented. Relative abundance was considered significant when P-adjusted value < 0.05.

**Supplementary Figure 6: Taxonomic analysis of the biofilm bacteria after thrombin exposure.** Mucosa associated microbiota (isolated from colonic biopsies) from healthy individuals (n=11), were cultured *in vitro* as a polymicrobial anaerobic biofilm and exposed to increasing concentration of human purified thrombin (ranging from 0 to 10 U/mL). DNA was extracted to seek for taxonomic differences after thrombin exposure. **(A)** The bar plots illustrate the relative abundance of the TOP12 taxa. Individuals are depicted as a summary bar plot for each condition corresponding to the mean of the relative abundance for each individual. **(B)** Overall diversity dissimilarity of fecal microbiota from each group was visualized using Principal Coordinate Analysis (PCoA) based on the weighted unifrac distance. PERMANOVA P-value >0.05 for all pairwise comparisons**.**

**Supplementary Figure 7: Taxonomic analysis of the biofilm bacteria after thrombin exposure (LEfSe).** Mucosa associated microbiota (isolated from colonic biopsies) from healthy individuals (n=11), were cultured *in vitro* as a polymicrobial anaerobic biofilm and exposed to increasing concentration of human purified thrombin (ranging from 0 to 10 U/mL). DNA was extracted to seek for taxonomic differences after thrombin exposure. Multigroup differential taxonomic analysis was performed using Linear discriminant analysis Effect Size (LEfSe), identifying taxa with significantly different relative abundances after 10U exposure of thrombin (adjusted P < 0.05; LDA score shown).

**Supplementary Figure 8: Taxonomic analysis of fecal microbiota from mice treated intracolonically with thrombin.** C57BL/6 mice were treated daily for 10 days via intracolonic instillation with either PBS (control - CTR, n = 5) or recombinant active thrombin (F2, 5 U/day, n = 5). At sacrifice, fecal samples were collected for 16S rRNA sequencing using Illumina (V3–V4 region). **(A)** Bar plots show the relative abundance of the top 5 most abundant phyla (top) and top 10 families (bottom) for each individual mouse in both groups. **(B)** Boxplots display alpha-diversity indices (Observed, Chao1, Shannon, and Fisher). Boxes represent interquartile range, and horizontal lines indicate the median. No statistically significant differences were observed (ANOVA with Tukey’s post-hoc test, P > 0.05). **(C)** Beta-diversity was assessed using Principal Coordinate Analysis (PCoA) based on weighted UniFrac distances; no significant clustering was detected (PERMANOVA, P > 0.05).

**Supplementary Figure 9: Taxonomic analysis of fecal microbiota from mice treated intracolonically with thrombin (LEfSe).** C57BL/6 mice were treated daily for 10 days via intracolonic instillation with either PBS (control - CTR, n = 5) or recombinant active thrombin (F2, 5 U/day, n = 5). At sacrifice, fecal samples were collected for 16S rRNA sequencing using Illumina (V3–V4 region). Differential taxonomic analysis was performed using Linear discriminant analysis Effect Size (LEfSe), identifying taxa with significantly different relative abundances (adjusted P < 0.05; LDA score shown).

**Supplementary Figure 10: FISH analysis of distal colon of mice treated intracolonically with Thrombin.** C57BL/6 mice were treated daily for 10 days via intracolonic instillation with either PBS (control - CTR, n = 5) or recombinant active thrombin (F2, 5 U/day, n = 5). **(A)** Representative images of tissue-associated microbiota in distal colon were obtained via 16S rRNA fluorescent in situ hybridization (FISH) in PBS- and thrombin-treated mice. Blue is DAPI staining for host nuclei, green is fluorescein-coupled wheat germ agglutinin for sugar-rich content (e.g., mucus layer), and red is the 16S-Cyanine3 probe for all bacteria. **(B)** Biofilm damage scores were quantified from 3–5 fields per mouse, with each dot representing the mean per mouse. Differences between two groups was assessed by Student’s t-test (P < 0.05 considered significant).

**Supplementary Figure 11: Shotgun metatranscriptomic profile of biofilm treated or not with human thrombin (Metacyc functions).** Mucosa associated microbiota (isolated from colonic biopsies) from healthy individuals (n=5) and Crohn’s disease (CD) patients (n=5), were cultured *in vitro* as a polymicrobial anaerobic biofilm. Biofilms from healthy individuals were exposed or not to 10 U/mL of human purified thrombin **(A)** Venn diagram represents shared and discordant metabolic pathways features found in each group. **(B-C)** Volcano plot showing the differential abundance (Wald test, p < 0.05) of metabolic pathways of biofilm-dispersed bacteria from **(B)** unexposed healthy controls (blue) and CD patients (red) and **(C)** thrombin exposed controls (green)**.**

**Supplementary Figure 12: Shotgun metatranscriptomic profile of biofilm treated or not with human thrombin (Enzyme Class EC functions).** Mucosa associated microbiota (isolated from colonic biopsies) from healthy individuals (n=5) and Crohn’s disease (CD) patients (n=5), were cultured *in vitro* as a polymicrobial anaerobic biofilm. Biofilms from healthy individuals were exposed or not to 10 U/mL of human purified thrombin **(A)** Venn diagram represents shared and discordant Enzyme Class (EC) features found in each group. **(B-C)** Volcano plot showing the differential abundance (Wald test, p < 0.05) of EC features of biofilm-dispersed bacteria from **(B)** unexposed healthy controls (blue) and CD patients (red) and **(C)** thrombin exposed controls (green)**.**

**Supplementary Figure 13: Taxonomic analysis of feces of thrombin-exposed donors and germfree mice recipient mice.** **(A)** Pooled fecal microbiota from mice after 10 days of intracolonically exposure to thrombin at 5U/mouse (5 U group, n= 7), 20U/mouse (20 U group, n= 8) or to saline (control - CTR group, n= 5). DNA was extracted to seek for taxonomic differences after thrombin exposure. The bar plots illustrate the relative abundance of the TOP5 phyla and TOP5 families. Individuals are depicted as a summary bar plot for each condition corresponding to the mean of the relative abundance for each individual. **(B)** Fecal microbiota from transplanted mice with fecal slurry from 5U group, 20U group and control group. The bar plots illustrate the relative abundance of the TOP5 phyla and TOP5 families. Individuals are depicted as a summary bar plot for CTR corresponding to the mean of the relative abundance for each individual and individually for the two experimental groups.

**Supplementary Figure 14: Thrombin-induced dysbiosis triggers inflammatory genes when inoculated into non-predisposed mice (heatmap).** C57Bl/6 axenic mice (6-8 weeks old) underwent oral gavage with colonic mucosa associated microbiota (fecal microbiota inoculation) from mice intracolonically exposed for 10 days to PBS intrarectal injection (CTR, n=5), human purified thrombin at 5 UI/mL (5U, n=7), human purified thrombin at 20 UI/mL (20U, n=8). Two mice were not exposed before sacrifice (True-GF, n=2). Four days after transplantation, colons were harvested for RNA extraction. The heatmap represents scaled relative expression of a targeted list of host response genes. The color gradient from blue to red correspond to scale relative mRNA expression, and we added a left-side color-coded annotation to classify genes according to the functional groups listed in the legend

**Supplementary Figure 15: Thrombin-induced dysbiosis triggers inflammatory genes when inoculated into non-predisposed mice (Principal Component Analysis).** C57Bl/6 axenic mice (6-8 weeks old) underwent oral gavage with colonic mucosa associated microbiota (fecal microbiota inoculation) from mice intracolonically exposed for 10 days to PBS intrarectal injection (CTR, n=5), human purified thrombin at 5 UI/mL (5U, n=7), human purified thrombin at 20 UI/mL (20U, n=8). Two mice were not exposed before sacrifice (True-GF, n=2). Four days after transplantation, colons were harvested for RNA extraction. Gene expression dataset was ordinated using Principal Component Analysis (PCA) with its corresponding bi-plot vectors. Pairwise PERMANOVA revealed statistical differences between the CTR group and 5U group (P<0.005), and 20U group respectively (P<0.005).

**Supplementary Figure 16: Thrombin-induced dysbiosis triggers inflammatory genes when inoculated into non-predisposed mice (Selected genes)**  C57Bl/6 axenic mice (6-8 weeks old) underwent oral gavage with colonic mucosa associated microbiota (fecal microbiota inoculation) from mice intracolonically exposed for 10 days to PBS intrarectal injection (CTR, n=5), human purified thrombin at 5 UI/mL (5U, n=7), human purified thrombin at 20 UI/mL (20U, n=8). Two mice were not exposed before sacrifice (True-GF, n=2). Four days after transplantation, colons were harvested for RNA extraction. Scatter plot depicts relative mRNA expression of **(A)** *PAR1*, **(B)** *PAR4,* **(C)** *LRF5* and **(D)**  *ATG16L1* for each cohorts of mice (one dot = one mouse). Each values were normalized to the CTR group (Fold change mRNA vs CTR). Statistical significance was determined by ANOVA followed by Tukey’s for multiple comparisons, where P<0.05 was considered significant (* = p < 0.05, ** = p < 0.01, *** = p < 0.005).

**Supplementary Figure 17: Thrombin-induced TLR5 enhancement is not mediated by direct proteolytic cleavage of flagellins.** Mucosa-associated microbiota from healthy colonic biopsies were cultured in vitro as polymicrobial anaerobic biofilms and exposed (or not) to purified human thrombin (10 U/mL). After 24 hours, thrombin was removed by washing, and biofilm supernatants were collected and sterile-filtered. Normalized volumes of these supernatants were applied to the apical surface of HEK-Blue™ hTLR5 monolayers. After 4 hours of co-culture, supernatants were transferred to QUANTI-Blue medium, and TLR5 activity was quantified via spectrophotometry following overnight incubation. For each donor, results were normalized to the mean of the thrombin-free condition. **(A)** Mean TLR5 activity from five donors exposed to 0 or 10 U/mL thrombin. **(B)** Filtered supernatants were further exposed (or not) to thrombin *in vitro*. TLR5 activity is shown for three thrombin-unexposed and five thrombin-exposed microbiota-derived supernatants. Statistical significance was determined by ANOVA followed by Dunnett’s for multiple comparisons, where P<0.05 was considered significant.

**Supplementary Figure 18: Thrombin has no major effect on E Coli biofilm structure and virulence. (A-B)** Several E. coli strains (LF82 wild-type, LF82 ΔfliC, and NRG) were cultured *in vitro* as a polymicrobial anaerobic biofilm and exposed to increasing concentration of human purified thrombin (ranging from 0 to 10 U/mL). **(A)** The total biofilm biomass was measured. **(B)** The rate of biofilm-dispersed bacteria was measured during a 24-hour period of culture. For each individual, raw values were normalized to the average values of the condition without thrombin. The plot represents the mean values for all replicates for each strain in each condition (human purified thrombin concentrations ranging from 0 to 10 U/mL). **(C)** *E. coli*/*Enterobacteriaceae* isolates were obtained from 3 patient-derived mucosal microbiota using RAPID'E.coli2 and cultured *in vitro* as a polymicrobial anaerobic biofilm and exposed or not to human purified thrombin (10 U/mL). Normalized counts of biofilm-dispersed bacteria were then cultured on the apical surface of the human HEK-Blue™ hTLR5 monolayer. After a 4-hour coculture, some supernatant was collected and applied to QUANTI-Blue medium. TLR5 activity was measured with spectrophotometric assay after overnight exposure. For each individual, raw values were normalized to the average values of the condition without thrombin. The plot represents the mean values for all individuals in each condition. Statistical significance was determined by ANOVA followed by Dunnett’s for multiple comparisons; no statistically significant differences were observed (all p > 0.05).
