# Supplementary material for: High thrombin activity associated with crohn’s disease induces microbiota pathogenicity contributing to mucosal inflammation
Source: Gut Microbes. 2026 Jun 17;18(1):2687903. doi: 10.1080/19490976.2026.2687903 (PMC13285544; doi:10.1080/19490976.2026.2687903)
Supplement: REVISED_suppFigures_ADOBE.pdf [file KGMI_A_2687903_SM4602.pdf]

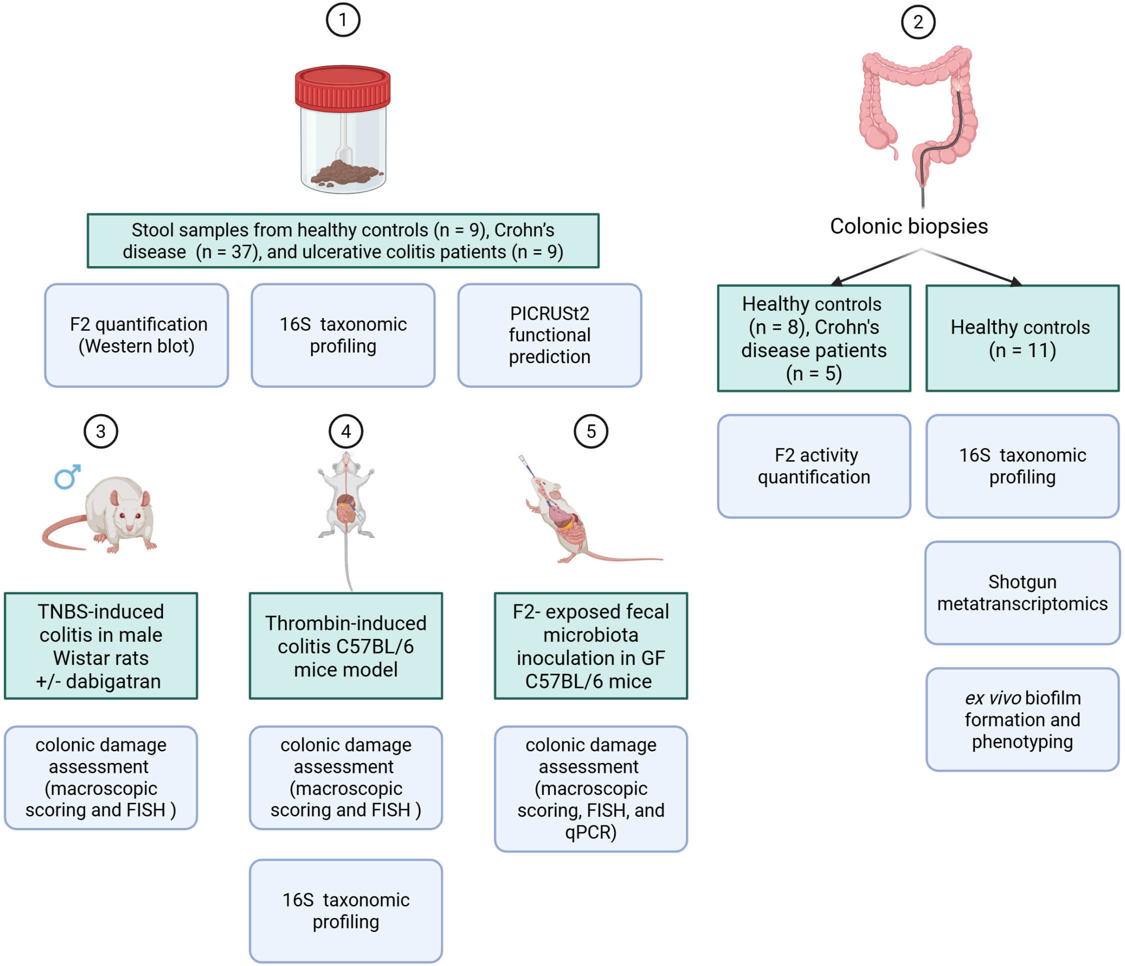

Supplementary Figure 1: Study design and experimental workflow.

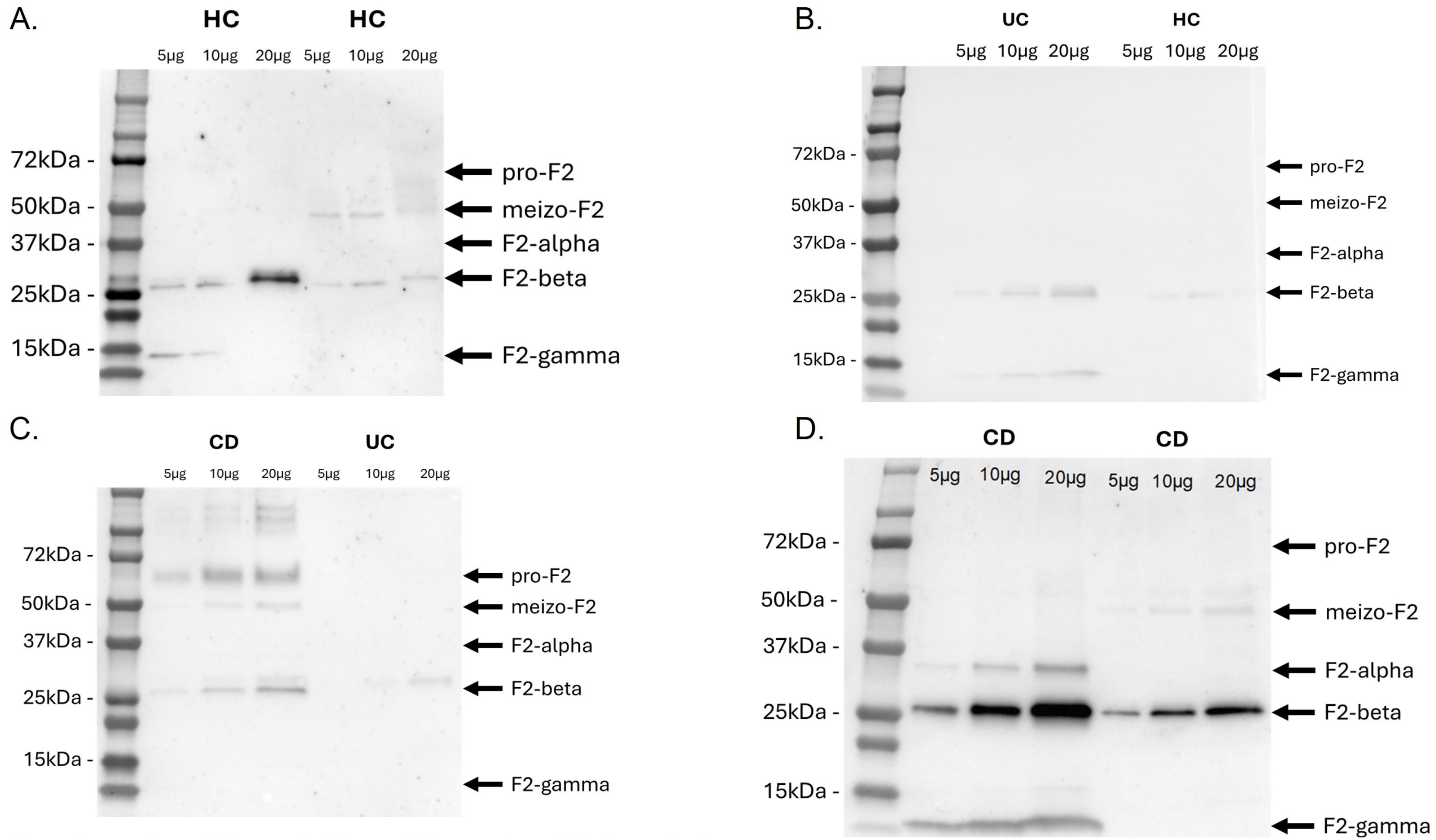

Supplementary Figure 2: Thrombin western blot analysis in human feces.

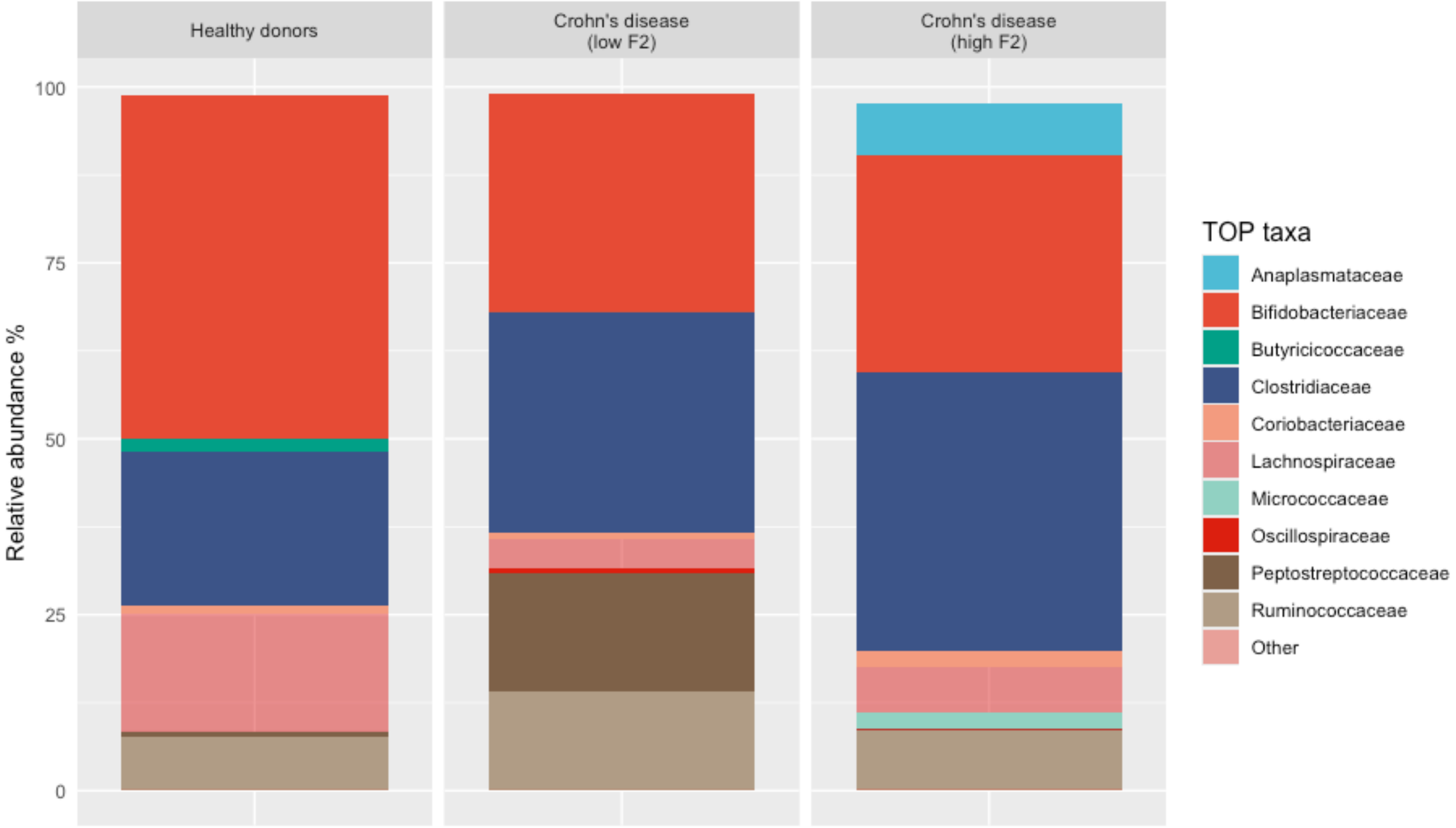

Supplementary Figure 3: Taxonomic analysis of feces from healthy donors and Crohn's disease (CD) patients (Grouped abundance).

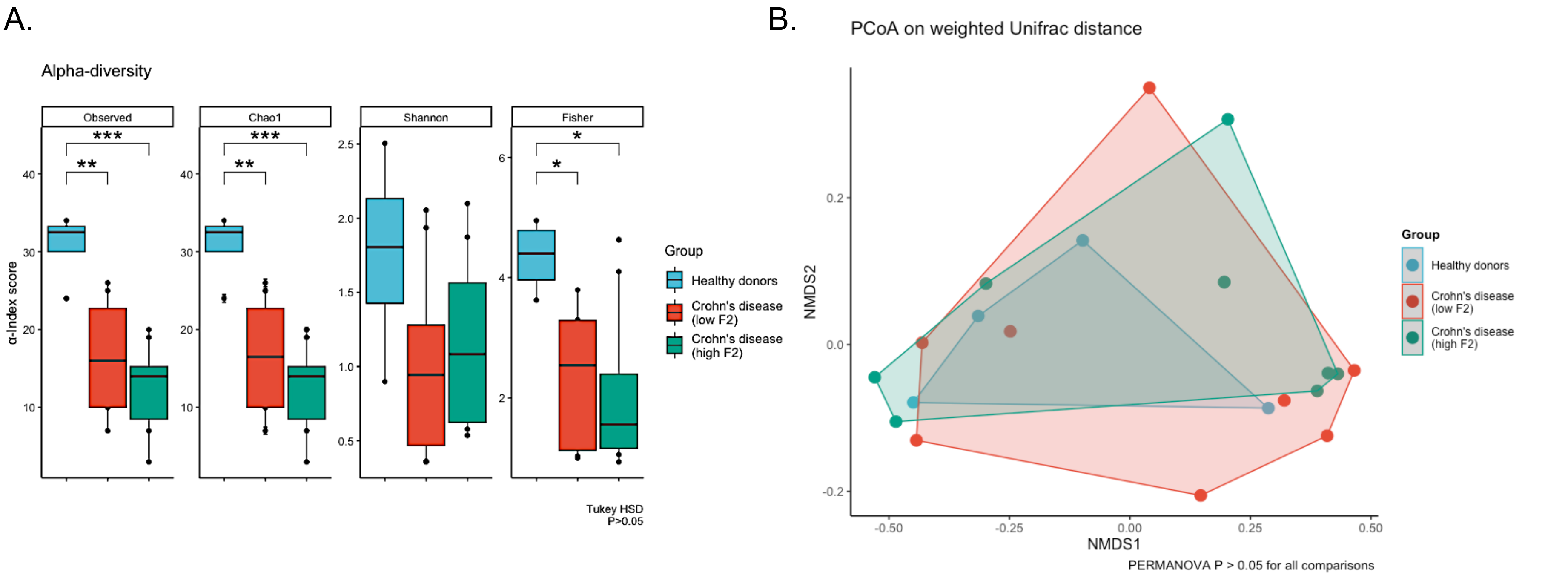

Supplementary Figure 4: Taxonomic analysis of feces from healthy donors and Crohn's disease (CD) patients (Alpha and Beta diversity).

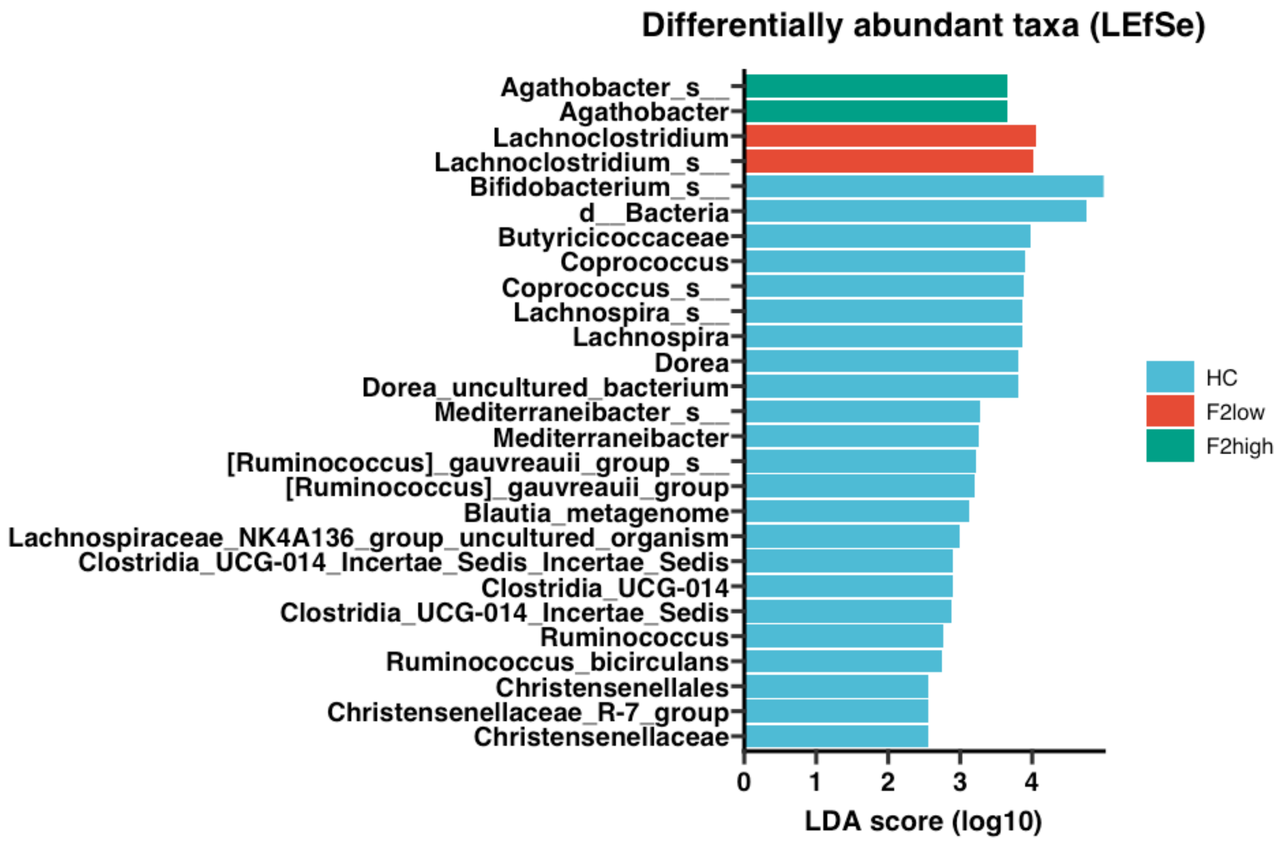

Supplementary Figure 5: Taxonomic analysis of feces from healthy donors and Crohn's disease (CD) patients (LEfSe).

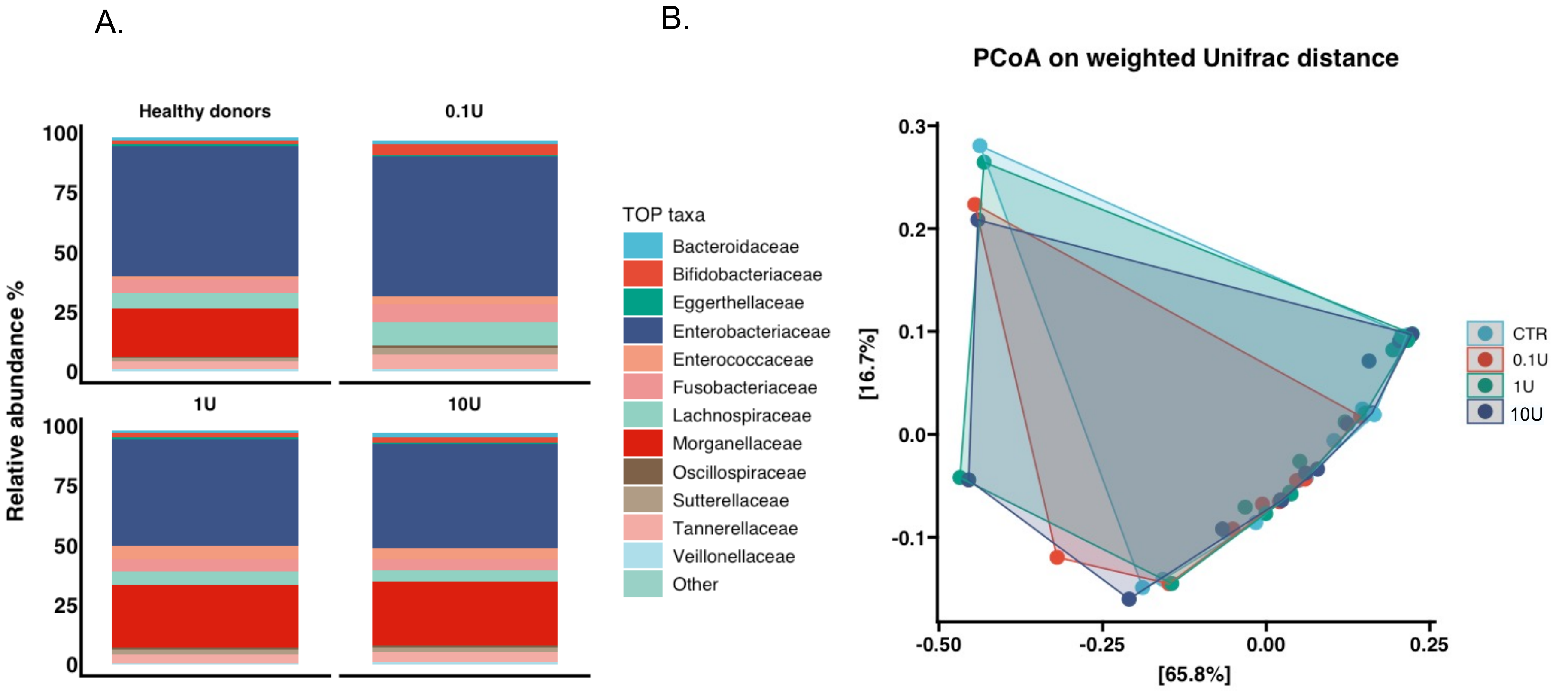

Supplementary Figure 6 Taxonomic analysis of the biofilm bacteria after Thrombin exposure.

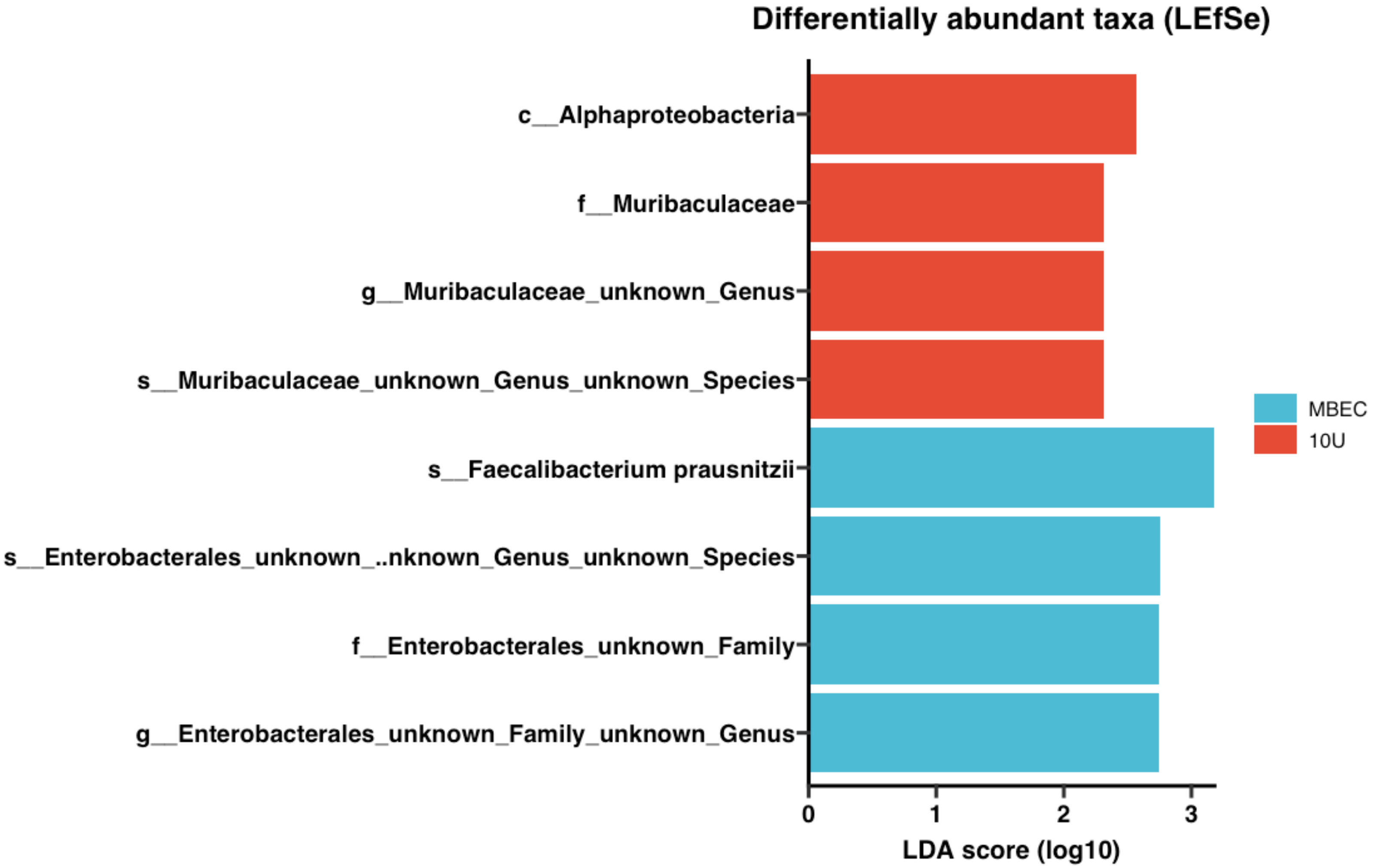

Supplementary Figure 7: Taxonomic analysis of the biofilm bacteria after Thrombin exposure (LEfSe).

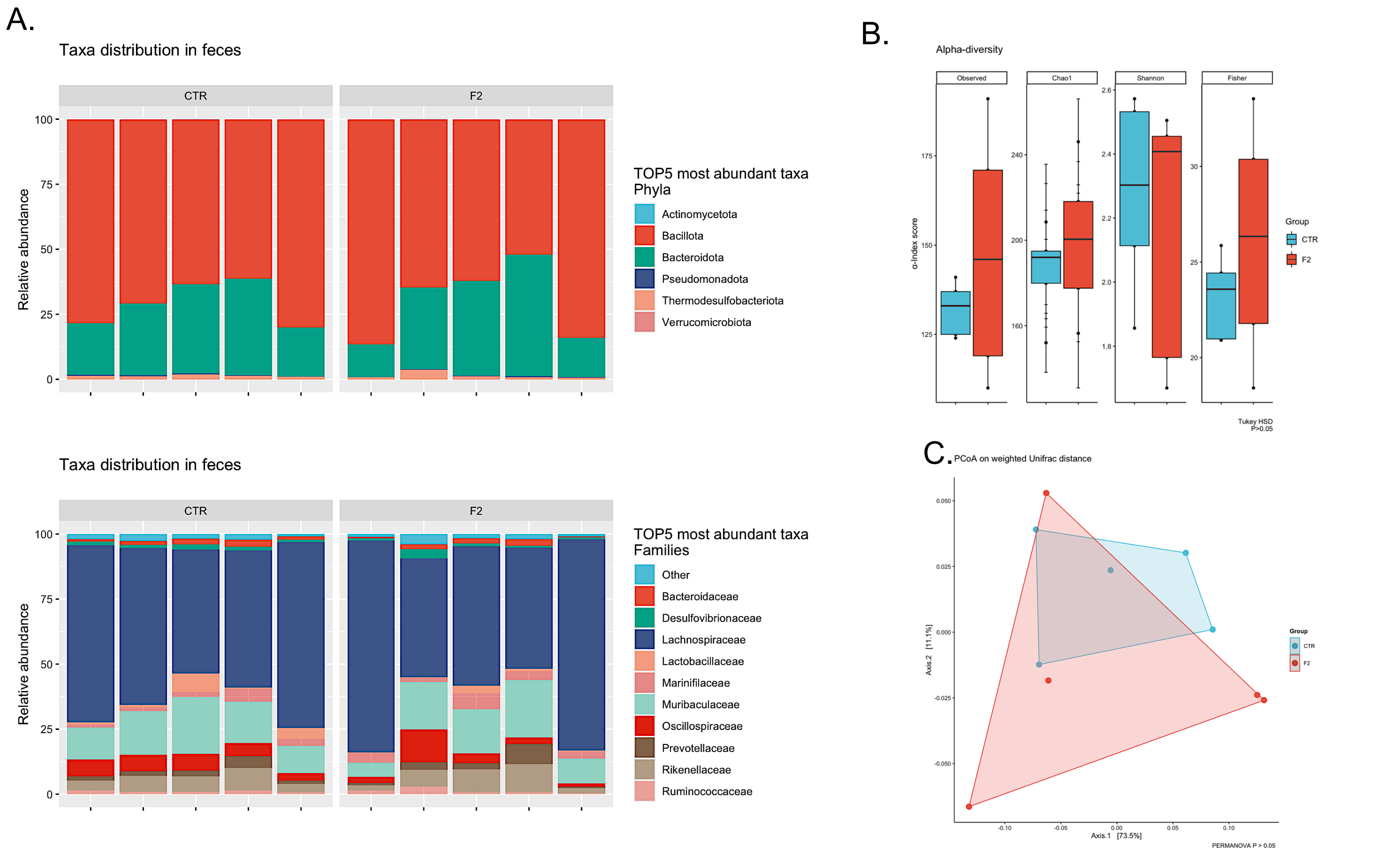

Supplementary Figure 8: Taxonomic analysis of fecal microbiota from mice treated intracolonically with Thrombin.

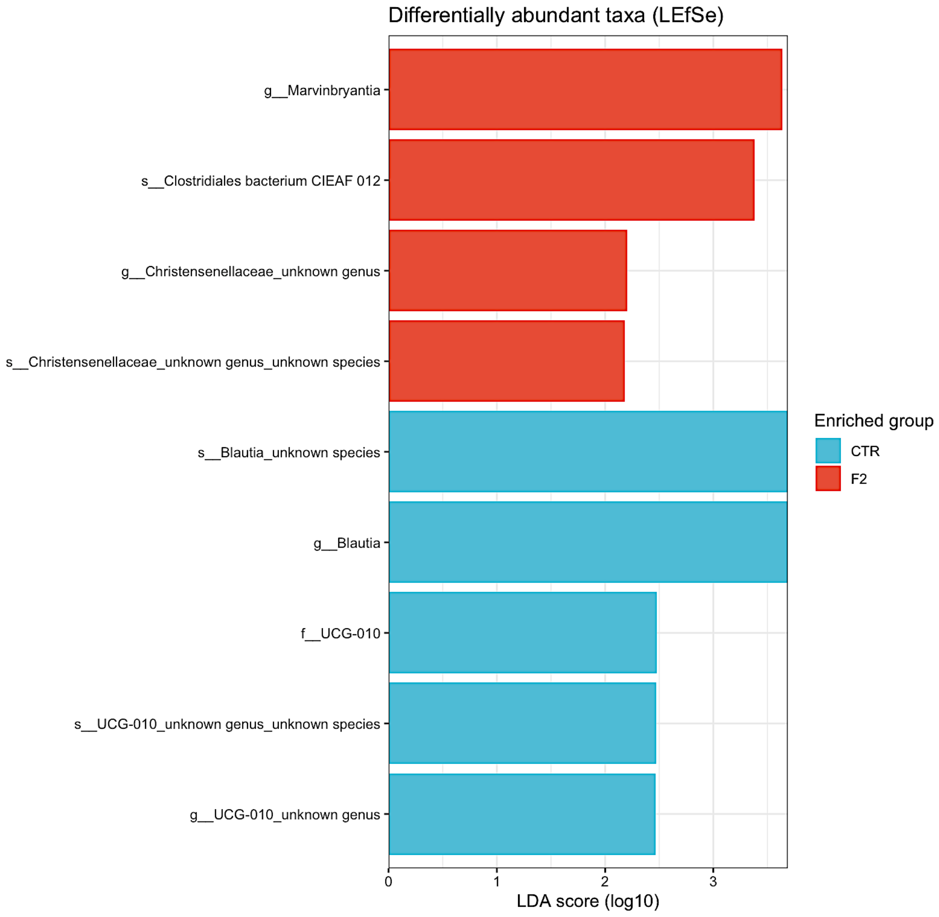

Supplementary Figure 9: Taxonomic analysis of fecal microbiota from mice treated intracolonically with Thrombin (LEfSe).

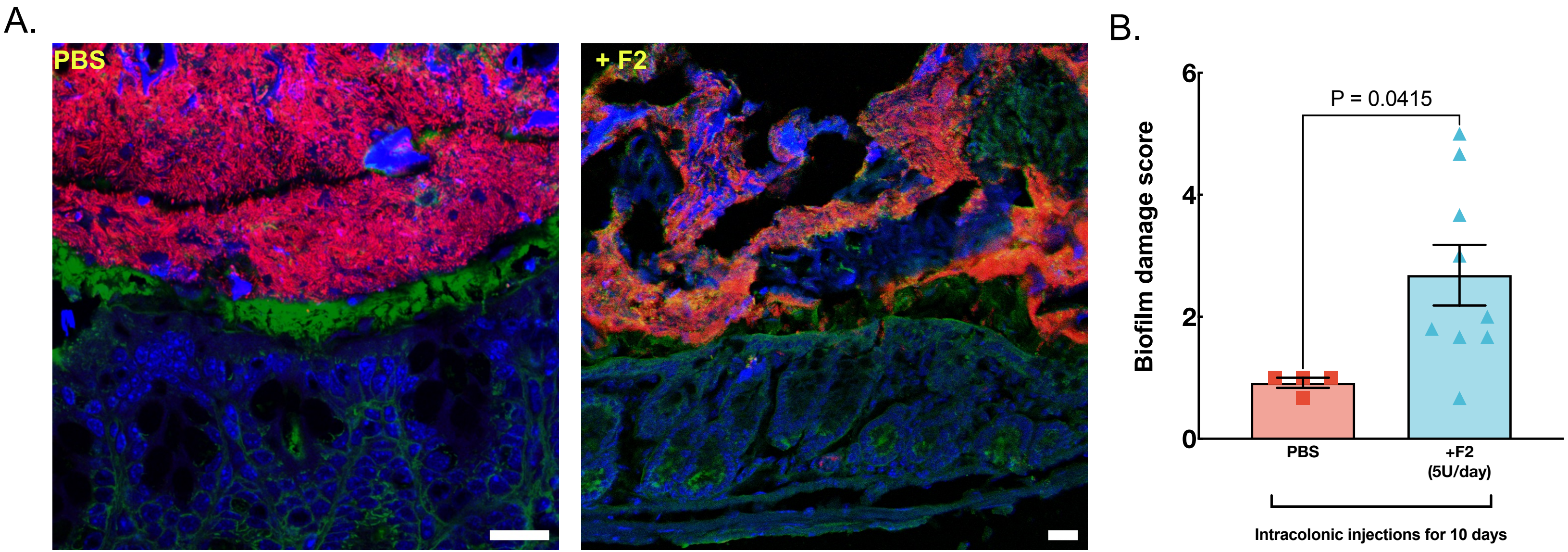

Supplementary Figure 10: FISH analysis of distal colon of mice treated intracolonically with thrombin.

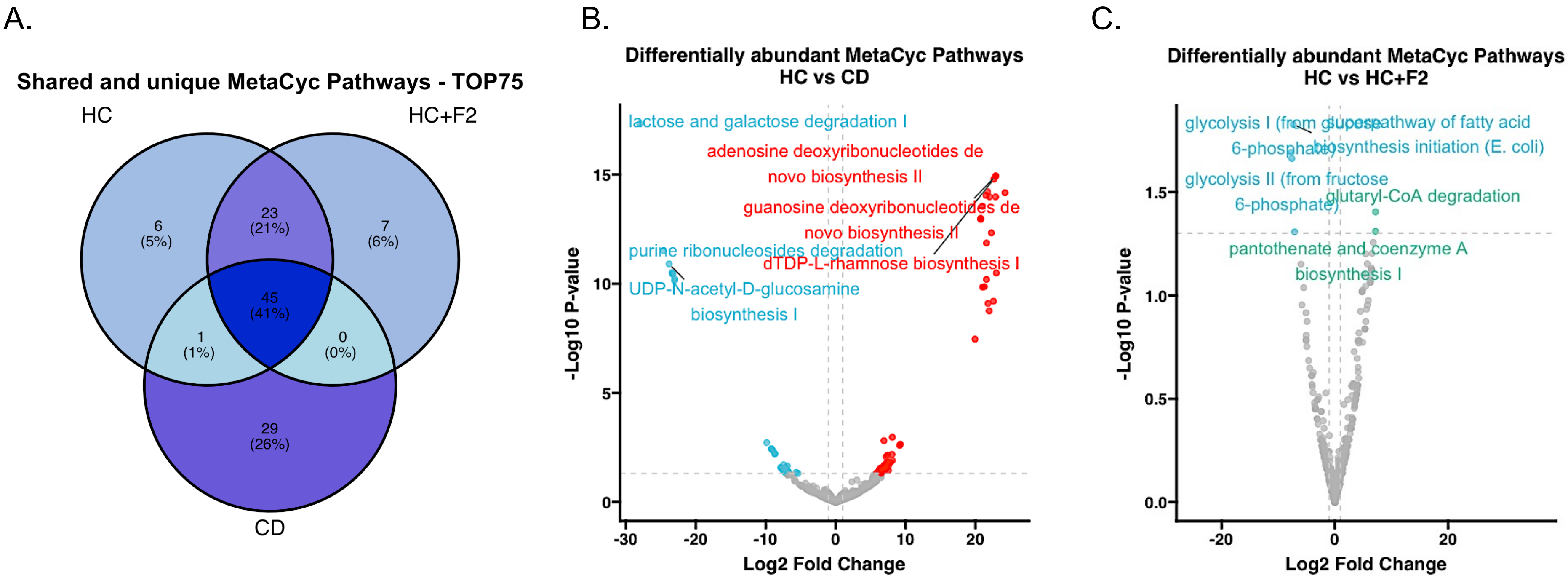

Supplementary Figure 11: Shotgun metatranscriptomic profile of biofilm treated or not with human thrombin (Metacyc functions)

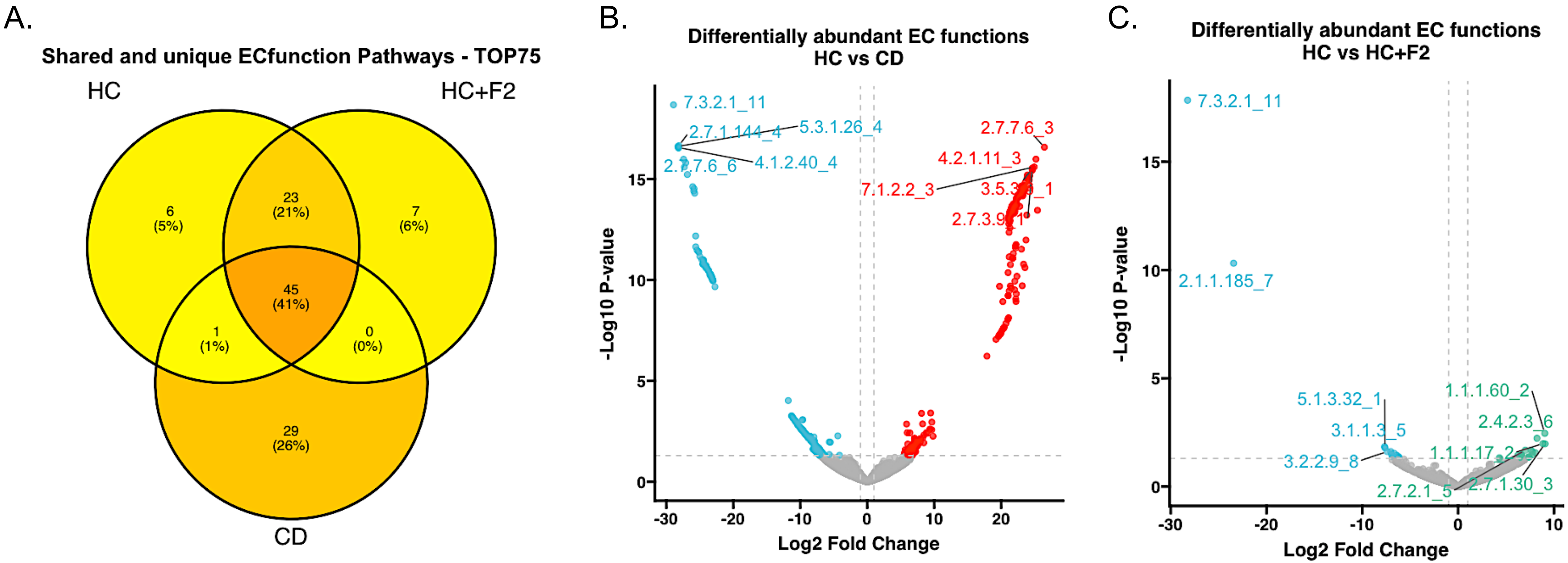

Supplementary Figure 12: Shotgun metatranscriptomic profile of biofilm treated or not with human thrombin (Enzyme Class EC functions)

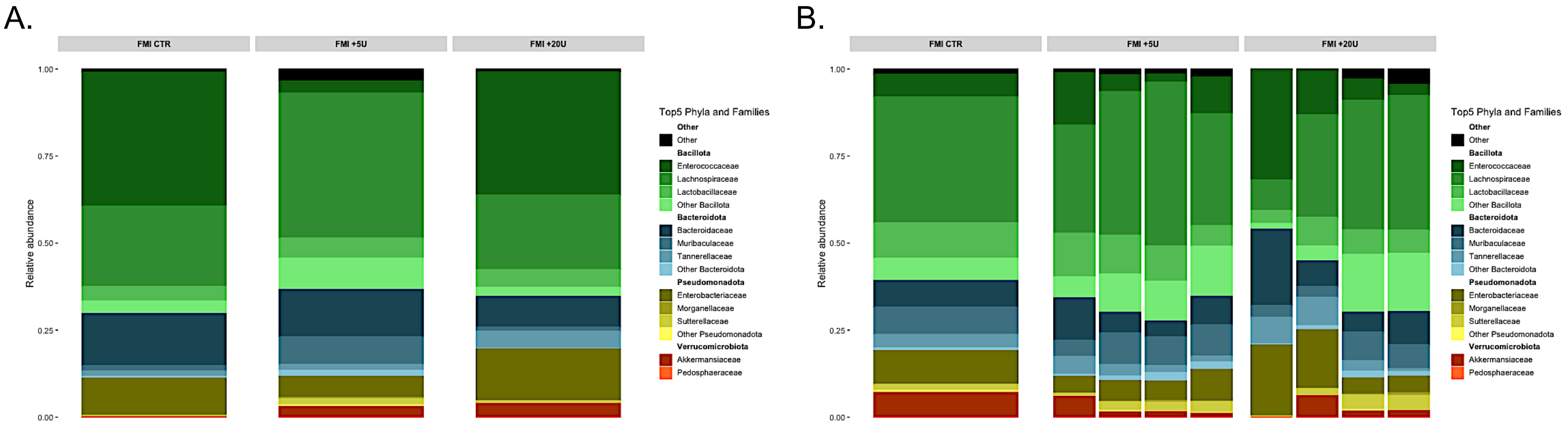

Supplementary Figure 13: Taxonomic analysis of feces of Thrombin-exposed donors and germfree mice recipient mice.

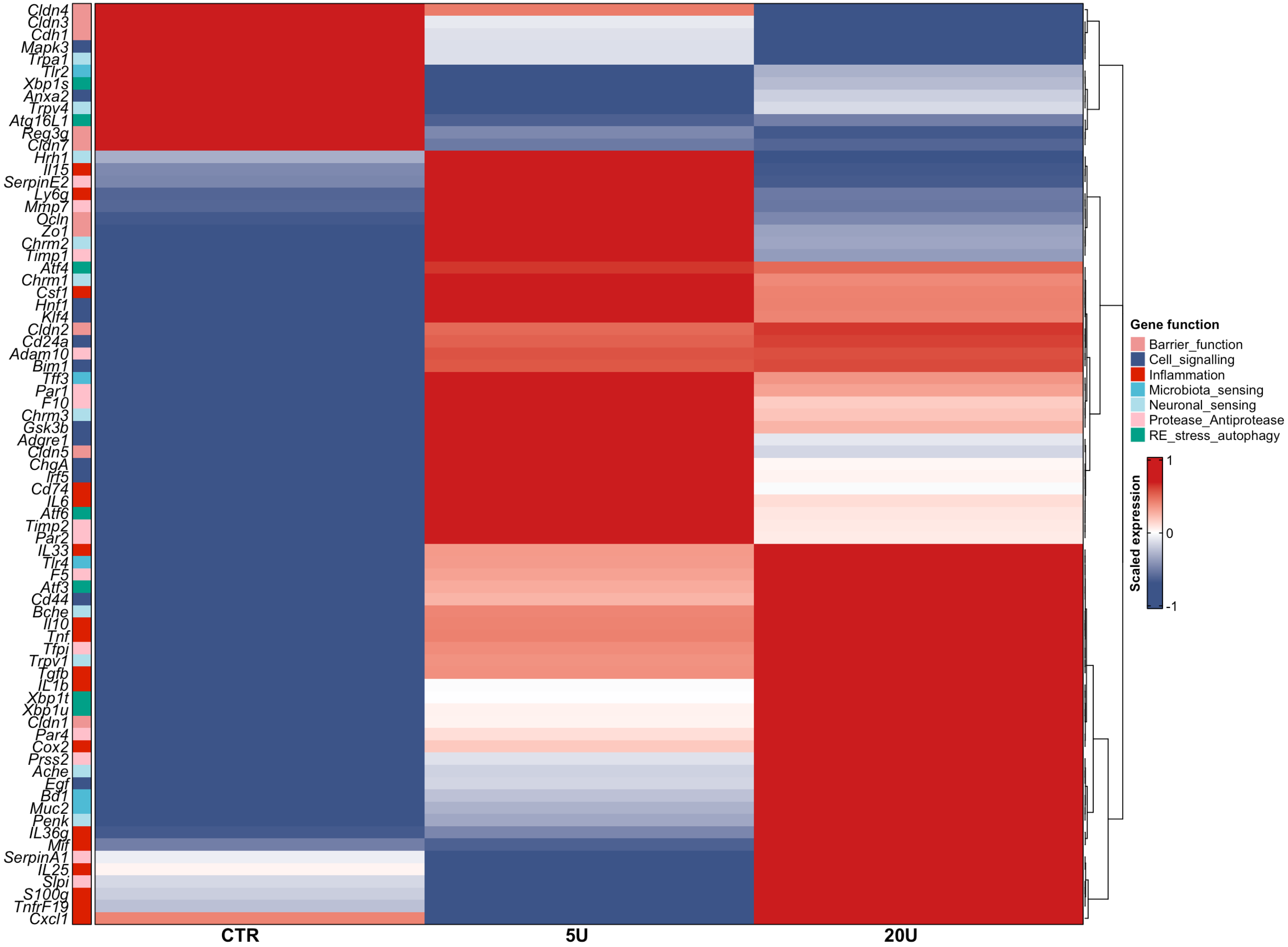

Supplementary Figure 14: Thrombin induced dysbiosis triggers inflammatory genes when inoculated into non-predisposed mice.

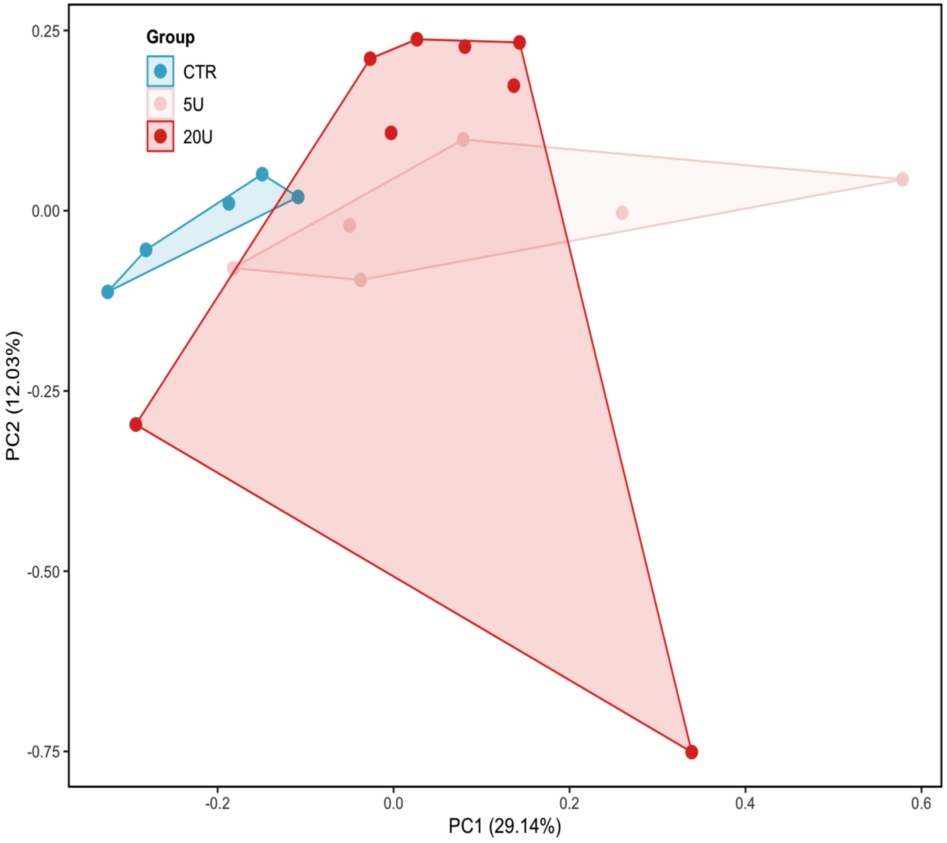

Supplementary Figure 15: Thrombin induced dysbiosis triggers inflammatory genes when inoculated into non-predisposed mice (Principal Component Analysis)

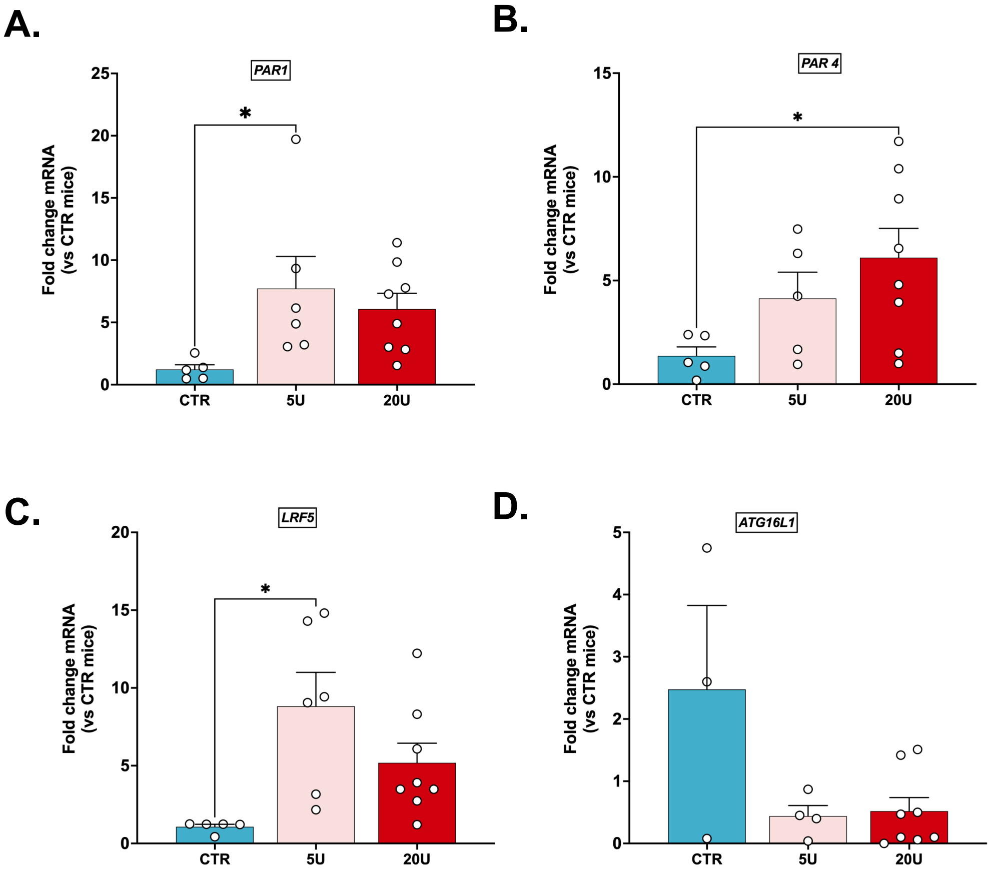

Supplementary Figure 16: Thrombin induced dysbiosis triggers inflammatory genes when inoculated into non-predisposed mice (Selected genes).

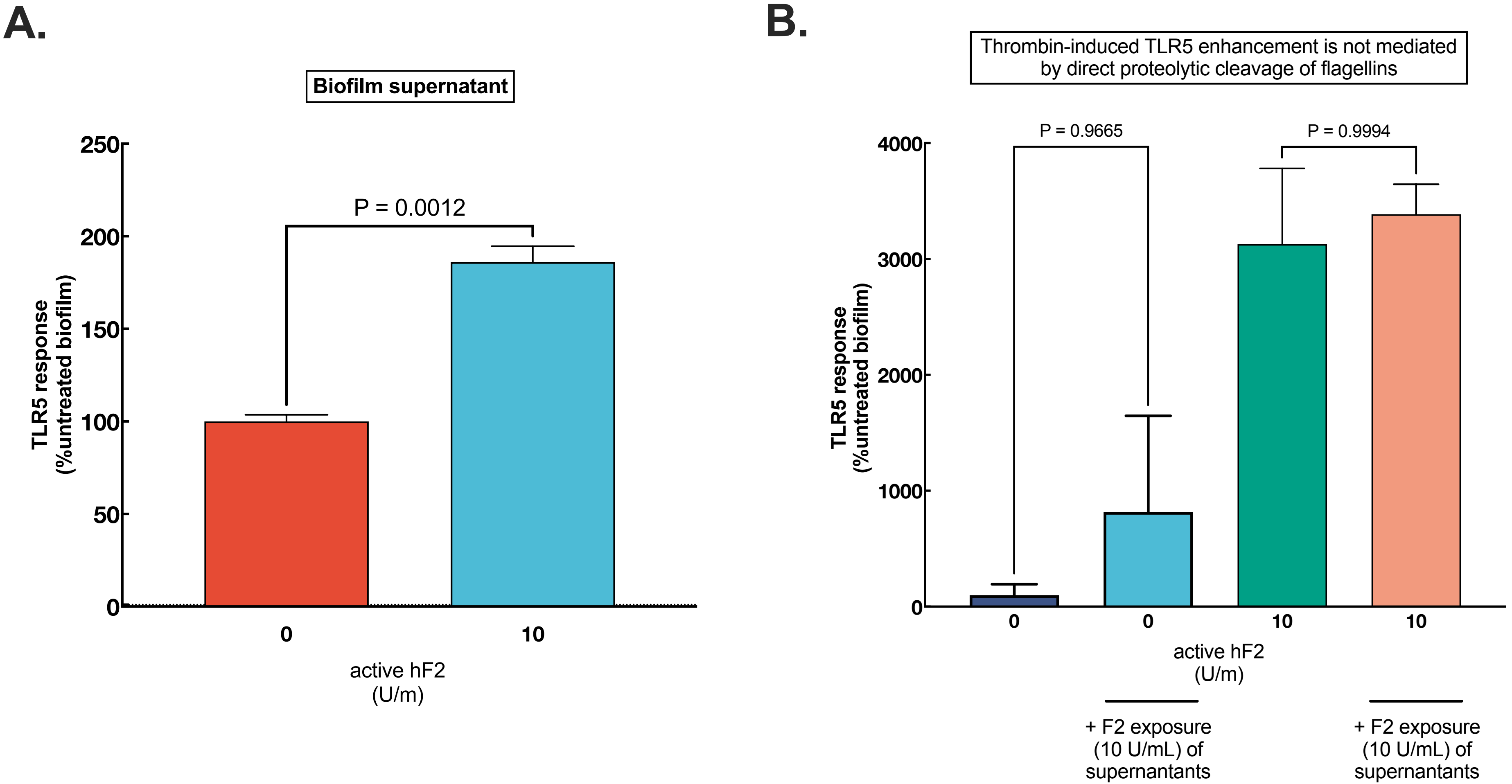

Supplementary Figure 17: Thrombin-induced TLR5 enhancement is not mediated by direct proteolytic cleavage of flagellins.

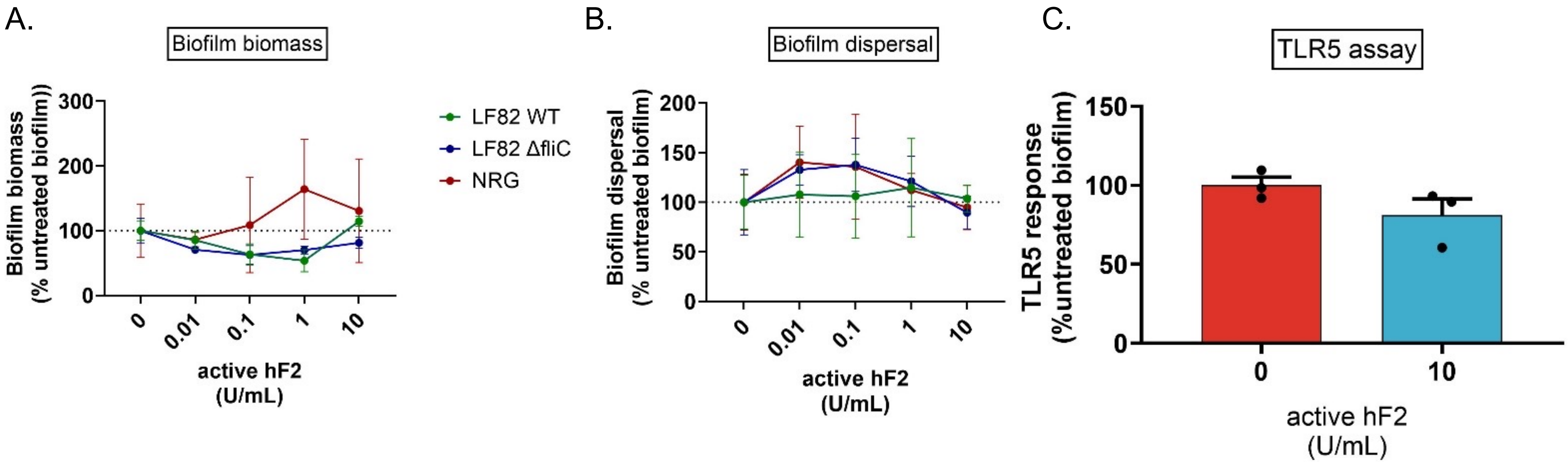

Supplementary Figure 18: Thrombin has no major effect on *E Coli* biofilm structure and virulence
